# *CSL* encodes a leucine-rich-repeat protein implicated in red/violet light signaling to the circadian clock in *Chlamydomonas*

**DOI:** 10.1371/journal.pgen.1006645

**Published:** 2017-03-23

**Authors:** Ayumi Kinoshita, Yoshimi Niwa, Kiyoshi Onai, Takashi Yamano, Hideya Fukuzawa, Masahiro Ishiura, Takuya Matsuo

**Affiliations:** 1 Center for Gene Research, Nagoya University, Nagoya, Japan; 2 Graduate School of Science, Nagoya University, Nagoya, Japan; 3 Graduate School of Biostudies, Kyoto University, Kyoto, Japan; Washington University School of Medicine, UNITED STATES

## Abstract

The green alga *Chlamydomonas reinhardtii* shows various light responses in behavior and physiology. One such photoresponse is the circadian clock, which can be reset by external light signals to entrain its oscillation to daily environmental cycles. In a previous report, we suggested that a light-induced degradation of the clock protein ROC15 is a trigger to reset the circadian clock in *Chlamydomonas*. However, light signaling pathways of this process remained unclear. Here, we screened for mutants that show abnormal ROC15 diurnal rhythms, including the light-induced protein degradation at dawn, using a luciferase fusion reporter. In one mutant, ROC15 degradation and phase resetting of the circadian clock by light were impaired. Interestingly, the impairments were observed in response to red and violet light, but not to blue light. We revealed that an uncharacterized gene encoding a protein similar to RAS-signaling-related leucine-rich repeat (LRR) proteins is responsible for the mutant phenotypes. Our results indicate that a previously uncharacterized red/violet light signaling pathway is involved in the phase resetting of circadian clock in *Chlamydomonas*.

## Author summary

The unicellular green alga *Chlamydomonas reinhardtii* is used as a model system in many biological researches. Although blue light responses of this alga (e.g., phototaxis) are well known and well characterized, far less is understood about responses to other wavelengths. One such photoresponse is the circadian clock, which can be reset by various wavelengths of light, ranging from violet to red, to entrain its oscillation to daily environmental cycles. In this study, we identified a gene responsible for red and violet light responses of the circadian clock by a forward genetic screen. Our results shed light on a previously unrecognized red/violet light signaling pathway in green algae.

## Introduction

Light is an important signal for most organisms, enabling them to interpret and respond to environmental conditions. In the green alga *Chlamydomonas reinhardtii*, various light responses are observed [1,2]. For example, blue and green light are sensed by channelrhodopsins, and regulate flagella activity for appropriate phototactic behaviors [3–5]. Blue light is also sensed by phototropins, and regulates sexual reproduction-related steps [6]. Moreover, red light regulates gene expression at the mRNA and protein levels [7–11]. Some responses to red light are mediated by aCRY, a homolog of animal cryptochromes, that can absorb not only blue light, but also a wide range of wavelengths, including red [11].

Circadian clocks are biological timekeepers that enable organisms to adapt to environmental daily cycles. The molecular mechanisms of the clocks have been studied in several model organisms, and "clock genes" have been revealed [12]. Clock genes are not conserved across the kingdom of organisms, but a common mechanism has been proposed [12]. In *Chlamydomonas*, several clock genes have been identified [13,14], and these genes revealed that the *Chlamydomonas* clock consists of land-plant-like and green-alga-specific components [15–18]. Because of its unique evolutionary position, *Chlamydomonas* can serve as an outstanding model organism for comparative and evolutionary studies of circadian clocks.

Although the circadian clocks can maintain their oscillation without any external cues, they need to be “reset” when a time lag between their oscillation and environmental daily cycles has occurred. A major cue that resets the circadian clock is light. In *Chlamydomonas*, the resetting in dark-adapted cells is induced by various wavelengths of light, ranging from violet to red [19,20]. A *Chlamydomonas* homolog of plant cryptochrome photoreceptors (CPH1) is known as a negative regulator for the resetting process by blue light [20]. In addition, aCRY is known to be involved in blue and red light responses of mRNA transcripts of several clock-related genes [11], although it remains to be determined to what extent these mRNA responses contribute to the phase resetting.

*RHYTHM OF CHLOROPLAST 15* (*ROC15*), a clock gene in *Chlamydomonas*, encodes a transcription factor-like protein with a GOLDEN2/ARR/Psr1 (GARP) DNA-binding motif. Mutations in *ROC15* affect period length and phase of the circadian clock [14]. Under the daily light/dark cycle, expression levels of ROC15 protein show a diurnal rhythm: ROC15 protein levels increase during the night, decline rapidly at dawn due to light-induced proteasomal degradation, and are kept low during the day [21]. Remarkably, red light is highly effective for degradation of ROC15, as well as the resetting of circadian clock [21]. In addition, since a *ROC15* gene mutant shows severe defect in the resetting of circadian clock by light, the light induced degradation of ROC15 is thought to be a key event for the resetting of the *Chlamydomonas* circadian clock [21]. However, photoreceptors and signaling pathways for this process remain unidentified.

Herein, we performed a forward genetic analysis to identify genes involved in ROC15 diurnal regulation, including the light induced degradation of the protein at the dawn. Through analyses of an isolated mutant, we show that at least two light signaling pathways are involved in the resetting of the circadian clock in *Chlamydomonas*, and one is a red/violet light signaling pathway wherein an uncharacterized *Chlamydomonas* leucine-rich repeat (LRR) protein is involved.

## Results

### Screening for mutants of ROC15 diurnal rhythm

**Fig 1:**
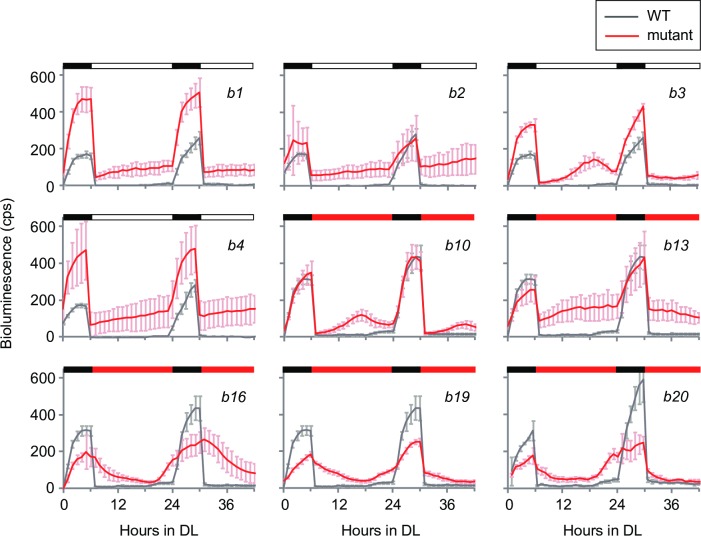
Diurnal rhythms of bioluminescence of ROC15-LUC in isolated mutants. Asynchronous TAP liquid cultures of mutants in white 96-well plates were subjected to two cycles of dark/light. White, red, and black bars represent white light, red light, and dark, respectively. Traces represent means ± standard deviation (SD) of bioluminescence levels from 4–6 independent cultures. A trace of the WT strain in the same assay is shown on each graph for comparison.

In a previous study, we developed a ROC15-LUC reporter strain that express a fusion protein of ROC15 and firefly luciferase to monitor fluctuations in ROC15 protein levels *in vivo* [21]. Taking advantage of the reporter strain and a high throughput bioluminescence monitoring system [22,23], we screened mutants showing abnormal diurnal rhythms through ROC15-LUC reporter bioluminescence. We employed a light regime consisting of two cycles of 6-h dark/18-h light. Under this regime, bioluminescence of the ROC15-LUC reporter increased during the 6 h dark period, declined immediately after light on, and was maintained at low levels throughout the 18 h light phase (S1 Fig, Wild-type [WT]). *roc114-1*, a clock mutant involved in the light induced degradation of ROC15 [21], showed an abnormal bioluminescence pattern (S1 Fig), indicating that this regime is appropriate for isolating mutants of ROC15 regulation. Insertional mutants were generated by random integration of a hygromycin-resistance gene (*aph7”*) [24] into the genome of the reporter strain. We screened 6,532 transformants under the regime, using white light as a light source for the day phase, and isolated 4 mutants, *b1* –*b4* (Fig 1). We further screened 7,672 transformants using red light as the day phase light, and isolated 5 mutants, *b10*, *b13*, *b16*, *b19*, and *b20* (Fig 1).

These nine mutants could be roughly divided into three types, 1–3. Type 1 (*b1*, *b2*, *b4*, and *b13*) showed insufficient decline of bioluminescence at light onset, and bioluminescence levels were maintained at relatively high levels during the light phase (Fig 1). This phenotype suggests that ROC15 synthesis or degradation is constantly high or low, respectively, in these mutants. Type 2 (*b3* and *b10*) showed almost normal acute response at the light onset but failed to maintain a low bioluminescence level during the mid to late day phase (Fig 1), suggesting the existence of a phase-specific mechanism for maintaining low ROC15 levels. Type 3 (*b16*, *b19*, and *b20*) showed no or very weak acute responses at light onset (Fig 1), suggesting defects in the light-induced rapid degradation of ROC15. The acute response was almost completely absent in *b16* in both the first and second cycles, but tended to be stronger in the second cycle compared to the first in *b19* and *b20* (Fig 1), indicating that *b19* and *b20* phenotypes, but not *b16*, are conditional.

### Red and violet light responses of ROC15 are impaired in *b16* mutant

**Fig 2:**
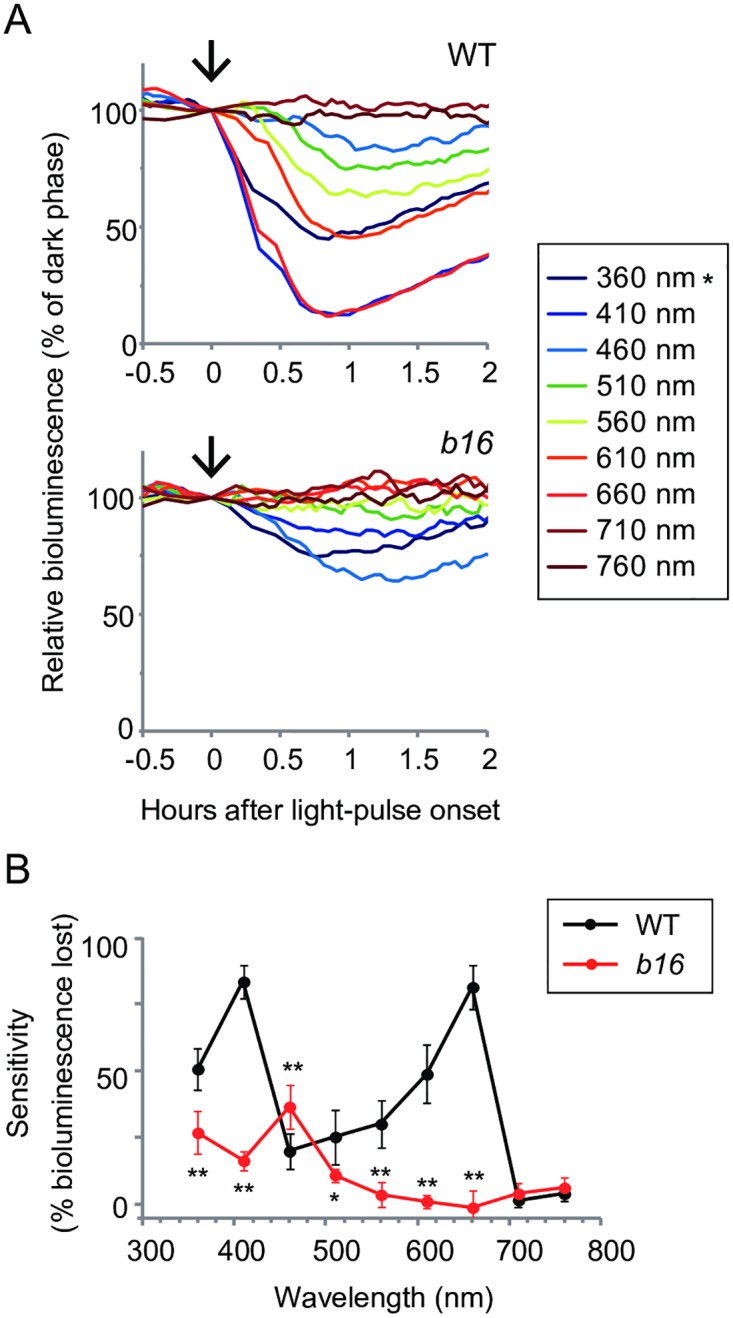
Wavelength dependency of the acute light response of ROC15-LUC. Asynchronous TAP cultures of WT and *b16* cells in black 24-well plates were subjected to darkness for 3 h for accumulation of ROC15-LUC, and then light pulses were administered. **(A)** Representative traces of the response to various light qualities. 5 min light pulses were administered by a tunable monochromatic light source. Each monochromatic light was given at 0.5 μmol∙m^-2^∙s^-1^ at the surface of the culture plates (arrows). Note that the 360 nm light (*) was weaker than those of other wavelengths at the sample level because the lid of culture plates blocked 30% of 360 nm light. The bioluminescence level just before light pulse was set to 100. **(B)** Sensitivities of ROC15-LUC to light. The sensitivity was represented as the relative bioluminescence level lost after light pulse at the time point when the minimum bioluminescence was detected. Each point represents the mean ± SD of 4–6 independent cultures. * *P* < 0.05, ** *P* < 0.01 (Student’s *t*-test).

To test whether the ROC15 acute light response phenotypes of *b16*, *b19*, and *b20* are dependent upon light wavelength, these mutants were subjected to blue or red light pulses, and ROC15-LUC bioluminescence was measured. Although *b19* and *b20* showed similar defects for both light sources, *b16* showed a different response (S2 Fig): Consistent with the observation in Fig 1, *b16* showed no acute response to the red light pulse (S2 Fig). However, the bioluminescence level declined to the same extent as the WT in response to the blue light pulse (S2 Fig). To obtain more detailed characteristics of wavelength dependency, we compared an equal-quantum action spectrum of the acute response of ROC15-LUC in *b16* mutant with that in WT. In WT strain, the light response of ROC15 was induced by blue (460 nm), green (510 nm), and especially red (660 nm) light pulses as we previously reported (Fig 2) [21]. In addition, we found that the light response of ROC15 was strongly induced by violet (410 nm) and ultraviolet (360 nm) light pulses, but not by far-red light pulses (710 nm and 760 nm) in the WT strain (Fig 2). In the *b16* mutant, the response to red light pulse was almost completely abolished (Fig 2). In addition, the response to the violet light pulse was partially impaired (Fig 2). These results indicate that *b16* has a defect in a pathway transmitting red and partial violet light information. On the other hand, the response to the blue light pulse (460 nm) was nearly normal or slightly stronger than that of the WT (Fig 2). This indicates that the blue light signal is transmitted by the other pathway. Taken together, these results indicate that there are multiple pathways (at least two) for transmitting light information to ROC15, and one is a red/violet light signaling pathway. Furthermore, the sensitization to blue light in *b16* implies the existence of a crosstalk between these light signaling pathways. In the following experiment, we focused our attention on the *b16* mutant.

### Photic phase-resetting of the circadian clock is impaired in *b16*

**Fig 3:**
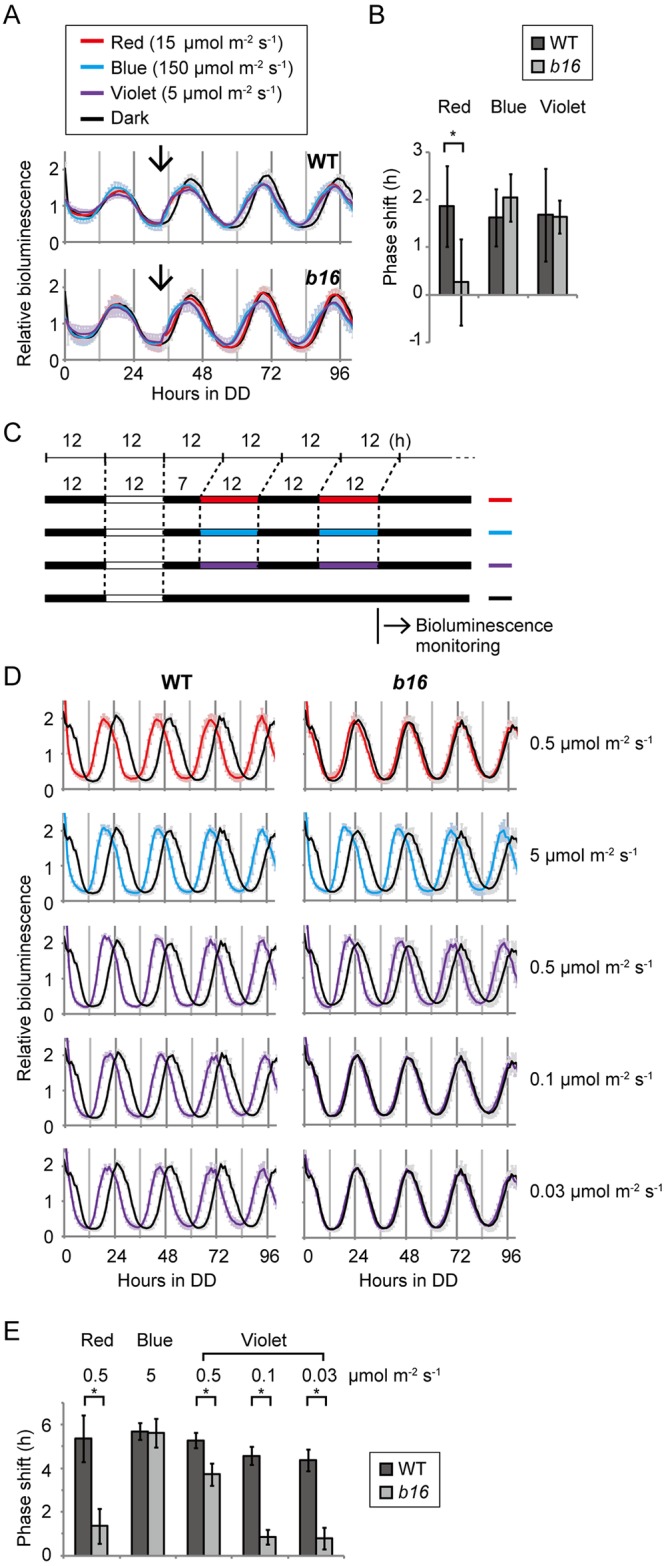
Phase resetting of the circadian clock in the *b16* mutant. Bioluminescence rhythms of 5-day-old spot cultures of WT and *b16* cells on HS agar media in white 96-well plates were measured. **(A)** Bioluminescence trace of a phase shifting experiment. Cells were exposed to a 12 h dark/12 h white light to entrain their circadian clock, and then bioluminescence rhythms were monitored in DD. 5 min light pulses were given at the 34 h time point in DD (arrows). Bioluminescence data are detrended by dividing by the 24 h moving average, and are shown as the mean ± SD of 10 independent cultures. **(B)** The amount of phase shift. Mean ± SD of the 10 independent cultures of the phase difference (h) between light-pulsed and dark control samples are shown. * *P* < 0.001 (Student’s *t*-test). **(C)** Schematic view of light conditions of the re-entrainment experiment. Cells were entrained by the first LD cycle of white light, and then re-entrained to a 5 h phase advanced LD cycles of red, blue, and violet light. Bioluminescence was monitored after release into DD. **(D)** Bioluminescence trace of re-entrainment experiment. Data are detrended by dividing by the 24 h moving average, and are shown as the mean ± SD of 10 independent cultures. **(E)** The amount of phase shift. The mean ± SD of 10 independent cultures of the phase difference (h) between light-entrained and dark control samples is shown. * *P* < 0.001 (Student’s *t*-test).

Because the rapid degradation of ROC15 by light has been suggested as a trigger for the phase-resetting mechanism in the circadian clock of *Chlamydomonas* [21], we tested photic phase-resetting in the *b16* mutant. For precise analyses of circadian rhythm, the ROC15-LUC reporter gene in *b16* was replaced by a genetic cross with the chloroplast *tufA* promoter-*lucCP* reporter gene of the CBR strain that shows a robust circadian bioluminescence rhythm [14,25]. Progenies harboring the *b16* mutation showed a normal circadian rhythm in the chloroplast bioluminescence reporter (S3A and S3B Fig), indicating that the *b16* mutation does not affect the circadian oscillation itself at least in constant darkness (DD). Next, we examined the effect of light pulses on the phase shift of the clock. Violet, blue, or red light pulse was given at the 34 h time point in DD (when the maximum phase advance occurs in this strain) [21]. Of all three light qualities tested, 1.6 to 1.9 h phase advances were observed in the WT (Fig 3A and 3B), whereas the amount of phase advance in *b16* was significantly reduced in response to the red light pulse (Fig 3A and 3B). There were no significant differences between WT and *b16* in the responses to blue and violet light pulse of these intensities tested (Fig 3A and 3B). Consistent with these results, the ROC15-LUC reporter strain showed a decline in bioluminescence in response to blue and violet but not red light in identical experimental conditions (S4 Fig).

In addition, we tested re-entrainment of the circadian rhythm. For this purpose, light/dark (LD)-entrained cells were subjected to two cycles of 5 h phase-advanced light/dark cycles of violet, blue, or red light, and then released into DD for determination of their circadian phase (Fig 3C). In the WT strain, the bioluminescence rhythm was advanced for 4.1 to 5.4 h compared to dark controls in all light qualities tested (Fig 3D and 3E), indicating that the circadian clock re-entrained to new LD cycles. However, re-entrainment was clearly inhibited in the *b16* mutant in red and weaker violet light conditions (Fig 3D and 3E). These results indicate that the phase-resetting of the circadian clock by red and violet light are impaired in the *b16* mutant.

### Light-induced phosphorylation of ROC15 is impaired in *b16*

**Fig 4:**
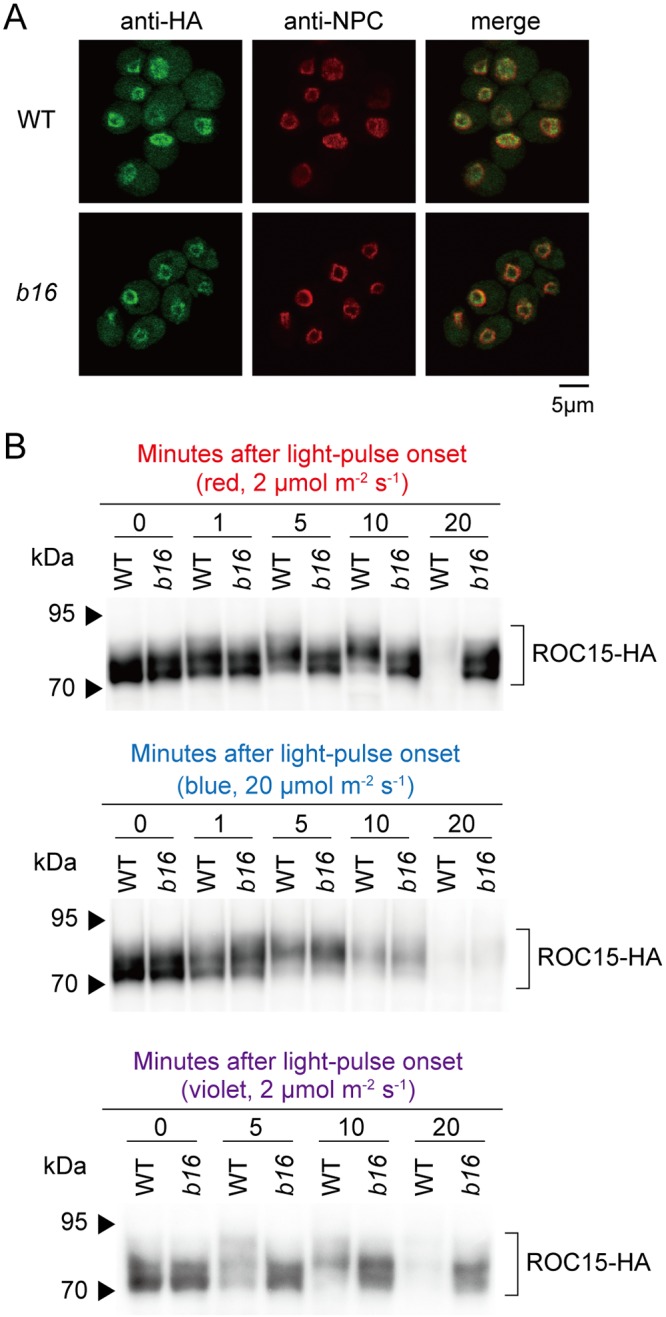
Subcellular localization and phosphorylation of ROC15. The *b16* strain (mt^+^) was genetically crossed with the ROC15-HA strain (mt^-^). A progeny harboring *b16* mutation and ROC15-HA transgene was selected by spot tests for antibiotic resistance and genomic PCRs. The hygromycin-resistance is genetically linked to *b16* (see S5 Fig). LD-synchronized cultures of the progeny in HS liquid medium were used. **(A)** Cells were harvested at midnight and subjected to immunocytochemistry. The nuclear membrane was counterstained using an antibody against the nuclear pore complex (NPC). Photographs obtained under the same settings of microscope and detector are shown. **(B)** Cells were exposed to a 0.5 min pulse of red (660 nm, 2 μmol∙m^-2^∙s^-1^), blue (470 nm, 20 μmol∙m^-2^∙s^-1^), or violet light (405 nm, 2 μmol∙m^-2^∙s^-1^) at midnight, and total protein extracts were subjected to Western blot analysis.

**Fig 5:**
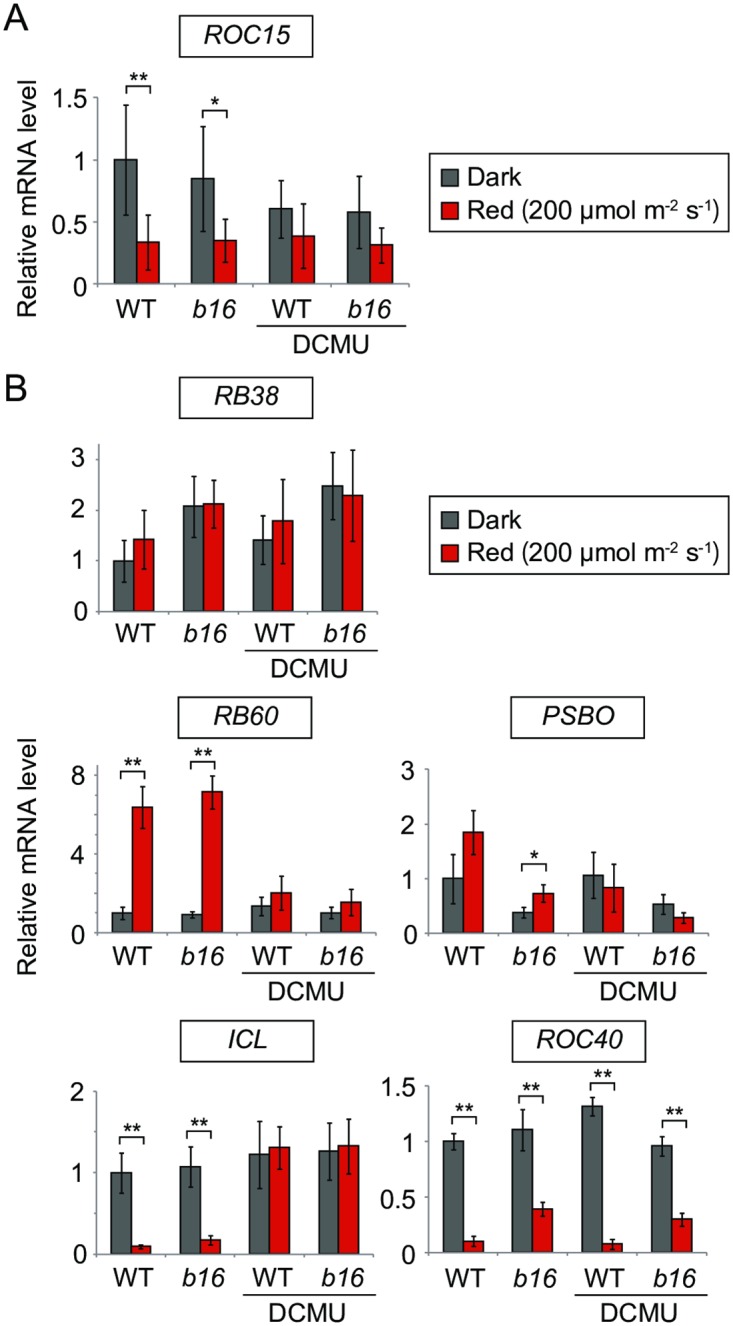
Red light response of *ROC15* and other mRNAs. Early log phase TAP liquid cultures (approximately 5 x 10^5^ cells/mL) of WT and *b16* were adapted to darkness for one day, and then exposed to red light (200 μmol∙m^-2^∙s^-1^) for 2 h. DCMU was added to the cultures before dark adaptation at a final concentration of 10 μM. **(A, B)** RT-qPCR result of *ROC15* (A) and other genes (B). Graphs represent the mean ± SD of 3–6 independent experiments. The transcript abundances relative to *RCK1* were further normalized by dividing by the average for the dark control of the WT for easy comparison. * *P* < 0.05, ** *P* < 0.01 (Student’s *t*-test).

Next, we tested the effect of *b16* mutation on several molecular aspects of ROC15. ROC15 is a nuclear protein that accumulates at night and is phosphorylated gradually in the darkness and is phosphorylated further after light exposure [21]. To analyze ROC15 in a *b16* background, we crossed *b16* with a ROC15-HA strain that expresses a hemagglutinin (HA) epitope-tagged ROC15 [21]. ROC15-HA was detected in the nucleus even in the *b16* background (Fig 4A), indicating that the *b16* mutation does not affect subcellular localization of ROC15. Western blot analysis of ROC15-HA from *b16* cells in the darkness detected a broad band, which is indicative of phosphorylated ROC15 [21] (Fig 4B, 0 min). However, no further mobility shift was observed in *b16* after red and violet light pulses (Fig 4B, 5 and 10 min). These results suggest that light-induced phosphorylation of ROC15, but not dark phosphorylation, is impaired in *b16*. After the blue light pulse, however, the further mobility shift was observed in *b16* as well as in the WT (Fig 4B). Taken together, these results indicate that *b16* has a defect in a red and violet light signaling pathway at a step prior to or at ROC15 phosphorylation.

In addition, the impaired ROC15-HA degradation in *b16* was evident at the 20 min time point of red and violet light pulsed cells (Fig 4B). This excludes the possibility that the *b16* phenotype in ROC15-LUC bioluminescence is an artifact caused by a defect in mechanisms related to bioluminescence.

### mRNA regulations of *ROC15* and other genes are essentially not affected by *b16*

The level of *ROC15* mRNA is downregulated by light [11,21]. Thus, we examined mRNA downregulation by red light in the *b16* mutant. As a result, decreased levels of mRNA transcripts were observed in *b16* (Fig 5A). This indicates that the light signaling pathway for *ROC15* mRNA downregulation is independent of *b16*.

In addition, there are several reports describing red light responses on the mRNA level. Among them, we tested three red light up-regulated genes (*RB38*, *RB60*, and *PSBO*) and two down-regulated genes (*ICL* and *ROC40*) [7,10,11]. As in previous reports, obvious increases in *RB60* and decreases in *ICL* and *ROC40* expression were observed in our experimental conditions (Fig 5B). 3-(3,4-dichlorophenyl)-1,1-dimethylurea (DCMU) blocked the response of *RB60* and *ICL* (Fig 5B), indicating that these responses are dependent on photosynthetic electron transport. Essentially the same red light responses were observed in the *b16* background (Fig 5B). These results indicate that the light signaling pathways for these genes are independent of *b16*. Although the decrease of *ROC40* mRNA level was weaker in *b16* (Fig 5B), *b16* mutation should not be responsible for this phenotype, as this phenotype was not rescued in a complemented strain of *b16* (see below).

Contrary to the previous report [10], little or no red-light-induction was observed in *RB38* and *PSBO* mRNA levels (Fig 5B). This may be due to differences in the duration of dark adaptation (1 day [this study] vs. 1 week [10]) or the timing of sample collection after light-on (2 h [this study] vs. within 1 h [10]).

### Identification of the disrupted gene in *b16*

**Fig 6:**
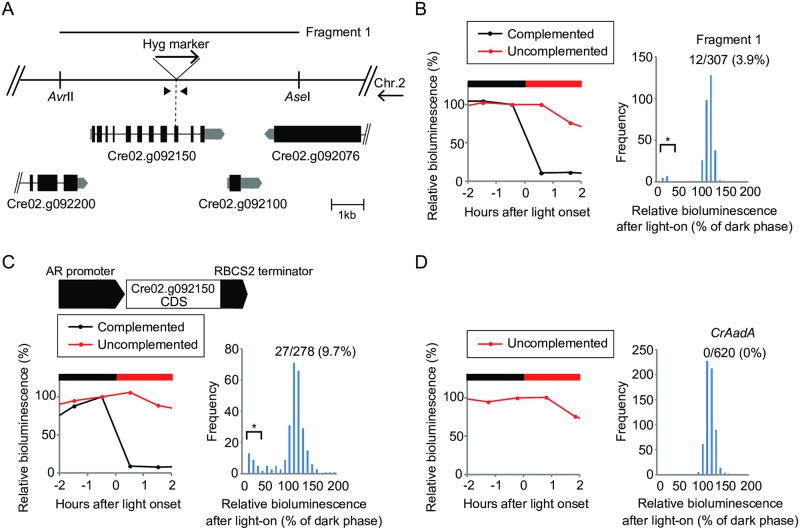
Identification of the disrupted gene in *b16*. **(A)** Schematic representation of genomic region around the insertion site (shown in reverse orientation of the sequence for chromosome 2). The specific primers bracketing the insertion site are shown as arrowheads. **(B**–**D)**, Complementation of the *b16* phenotype. *b16* mutant was transformed with a gDNA fragment (B, Fragment 1) and cDNA of the Cre02.g092150 gene (C). The left panels show ROC15-LUC bioluminescence of representative transformants monitored under the same conditions as Fig 1. Data before and after the light onset of the first DL cycle are shown. The bioluminescence level just before light onset was set to 100. The right panels are histograms representing the distribution of bioluminescence levels of all transformants after light on (relative to the dark phase). Asterisks indicate complemented transformants (the relative bioluminescence level after light on < 30). Numbers of complemented transformants and the rate of complementation are indicated in the graphs. A negative control experiment where *b16* mutant cells were transformed by *CrAadA* marker only is shown (D).

To identify the disrupted gene in *b16*, we first examined genetic linkage between the *b16* phenotype and hygromycin-resistance. Of progeny obtained in a genetic backcross to the parental strain, all hygromycin-resistant (Hyg^R^) and sensitive (Hyg^S^) progenies showed *b16* and WT phenotypes in the ROC15 light response, respectively (S5A Fig). In addition, Southern blot analysis of the marker gene in the genomic DNA (gDNA) of *b16* detected single bands (S5B Fig). These results indicate that *b16* has a single insertion of the marker gene causing the *b16* phenotypes. Thermal asymmetric interlaced PCR (TAIL-PCR) for the downstream of the marker gene amplified a DNA fragment (S5C Fig) corresponding to an uncharacterized gene on chromosome 2 (Cre02.g092150) (Fig 6A). PCR with a specific primer set bracketing the insertion site (Fig 6A, arrowheads) amplified a longer DNA fragment in *b16* than in the WT (S5D Fig), and direct sequencing analysis of the fragment revealed that the marker gene was inserted into an exon of Cre02.g092150 (*C*. *reinhardtii* ver. 5.5, Phytozome, Joint Genome Institute) [26] with a small (8 bp) deletion (Fig 6A, Hyg marker). A gDNA fragment containing this gene (Fig 6A, Fragment 1) almost fully complemented the red light response phenotype of *b16* (Fig 6B), and a WT transcript of Cre02.g092150 was restored in the complemented strains (S6 Fig). In addition, Cre02.g092150 cDNA driven by the *HSP70A*/*RBCS2* fusion promoter (AR promoter) [27] complemented the phenotype again (Fig 6C). As a negative control, we transformed the *b16* mutant with a selectable marker gene (*CrAadA*) alone and confirmed that no revertant appeared in this experimental condition (Fig 6D).

The Cre02.g092150 is annotated as a part of large single gene fused with two upstream genes (Cre02.g092200 and Cre02.g092250) in GenBank/EMBL/DDBJ (XP_001701983) [26], presumably by an automatic annotation (S7A Fig). Cre02.g092200 encoding a protein motif that putatively acts as a binding site for pyrroloquinoline quinone (PQQ), a known redox cofactor [28]. Although, to our knowledge, there is no evidence that PQQ acts as a cofactor for light signaling molecules, the existence of this motif encouraged us to search for longer variants fused with the upstream genes. Through RT-PCR analysis, we could detect a transcript corresponding to Cre02.g092150, but longer variants were undetectable at least under our PCR conditions (S7B Fig). Furthermore, the complementation rates of the *b16* phenotype were essentially identical between longer gDNA fragments covering the upstream genes and Cre02.g092150 gene alone (S7C Fig). These results indicate that Cre02.g092150 is sufficient to rescue the red light response of *b16*.

### Cre02.g092150 is responsible for other phenotypes of *b16*

In addition to the red light response of ROC15-LUC, we tested whether Cre02.g092150 can restore the other *b16* phenotypes or not. In the complemented strains (Fig 6B, S6 Fig), the weak response to violet light in ROC15-LUC bioluminescence (Fig 2) was also restored to the WT level at all light intensities tested (S8 Fig). To examine the re-entrainment phenotype (Fig 3C, 3D and 3E), the *b16* mutant carrying chloroplast bioluminescence reporter (S3 Fig) was transformed with the Fragment 1 (Fig 6A). Some transformants showed a normal re-entrainment phenotype (S9A and S9B Fig) to the 5 h advanced red light cycle (Fig 3C). In addition, the restoration of re-entrainment was observed in not only red light but also violet light in these complemented strains (S9C Fig). To test the phenotype of ROC15 phosphorylation, we first complemented the *b16* mutation by a FLAG-tagged version of Cre02.g092150 gene (Cre02.g092150-FLAG) (S10 Fig), and then the complemented strain was genetically crossed with a ROC15-HA strain [21] to obtain progenies that harboring the ROC15-HA gene and both of the *b16* mutation and Cre02.g092150-FLAG gene. The phosphorylation of ROC15-HA by red and violet light pulses was restored in this strain (S11 Fig), indicating genetic complementation of the phosphorylation phenotype of *b16* by Cre02.g092150-FLAG transgene. Taken together, these results indicate that Cre02.g092150 is responsible for almost all the *b16* phenotypes in the ROC15 light response. On the other hand, the weak downregulation of *ROC40* mRNA by red light (Fig 5B) was not rescued in the complemented strain (S12 Fig), suggesting that this phenotype is not caused by the mutation of Cre02.g092150 but rather by the genetic background of the strain.

### Cre02.g092150 encodes a LRR protein similar to RAS-signaling-related LRR proteins

**Fig 7:**
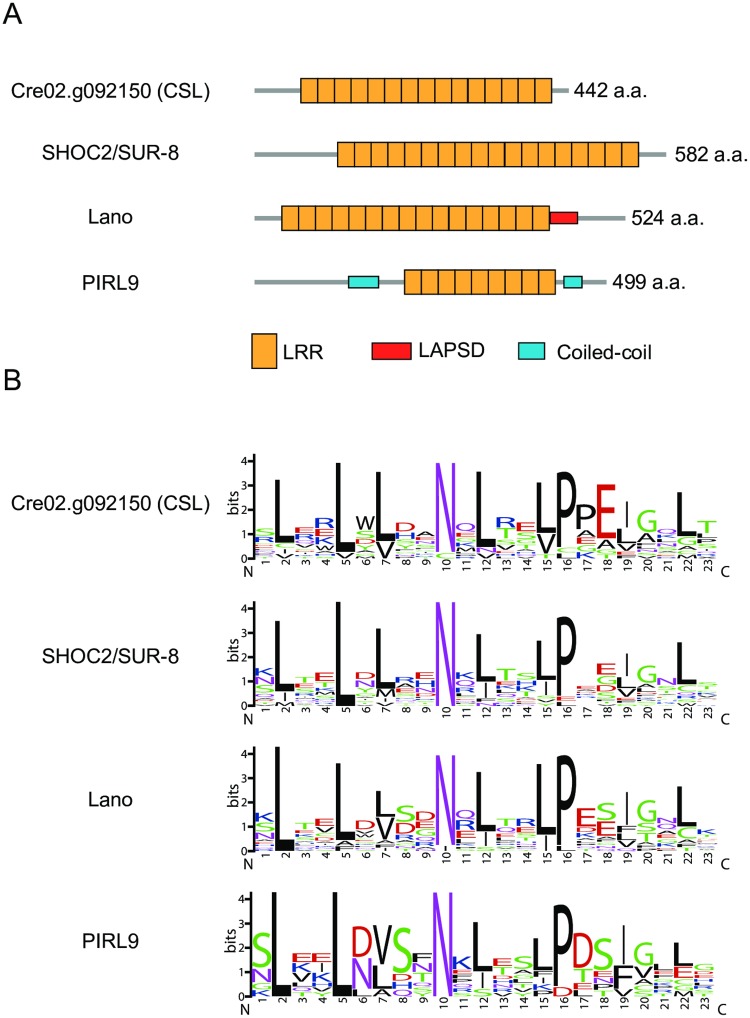
Sequence analysis of Cre02.g092150. **(A)** Schematic representation of domain architecture of Cre02.g092150 and other LRR proteins. PIRL9 is shown as a representative of the PIRL family. **(B)** Consensus LRR sequences visualized by the program WebLogo (http://weblogo.berkeley.edu/)[49].

Cre02.g092150 encodes a 442 amino acid protein comprised almost exclusively of LRR (Fig 7A). A homology search against protein sequences of major model organisms in GenBank/EMBL/DDBJ revealed that a hypothetical protein of Volvox *carteri*, a close relative of *Chlamydomonas*, was 76% identical to Cre02.g092150. In addition, many LRR proteins in a wide range of organisms, including animals and land plants, were detected, although their identities to Cre02.g092150 were low (< 40%). Among them, Cre02.g092150 showed relatively high similarity to animal SHOC2/SUR-8 proteins and LAP (LRR and PDZ [PSD-95/Disc Large/ZO-1]) family proteins [29–31], and their close relatives in land plants (PIRL [Plant Intracellular Ras-group LRR] family proteins) [32]. SHOC2/SUR-8 is known to act as a scaffold protein for interaction of RAS and RAF proteins [33]. In addition, LAP proteins are also known to associate with the SHOC2/SUR8-RAS complex [34,35]. SHOC2/SUR-8 and Cre02.g092150 are both composed almost extensively of LRRs [29,30] (Fig 7A). LAP family proteins have an N-terminal LRR domain followed by a LAP-specific domain (LAPSD) and a C-terminal PDZ domain, but Lano, a member of the family, lacks the PDZ domain [36] (Fig 7A). PIRL proteins have much shorter LRR domains and more extensive N-terminal regions [32] (Fig 7A). The LRR consensus amino acid sequences of these proteins, including Cre02.g092150, are highly similar (Fig 7B). The consensus sequences indicate that they belong to the plant-specific class of the LRR subfamily [37,38] but lack a glycine residue that is found in the extracellular LRR module of plant LRR-RLK (LRR Receptor-Like protein Kinase) family proteins [39] (Fig 7B, between positions 13 and 14). Collectively, these data suggest that the *Chlamydomonas* Cre02.g092150 protein is a close relative of RAS-signaling-related LRR proteins found in other organisms. Therefore, we hereafter refer to Cre02.g092150 gene and *b16* mutant as *CSL* (*Chlamydomonas* SHOC2/SUR8-like LRR) gene and *csl* mutant, respectively.

### CSL is constitutively expressed and localized to the cytoplasm

**Fig 8:**
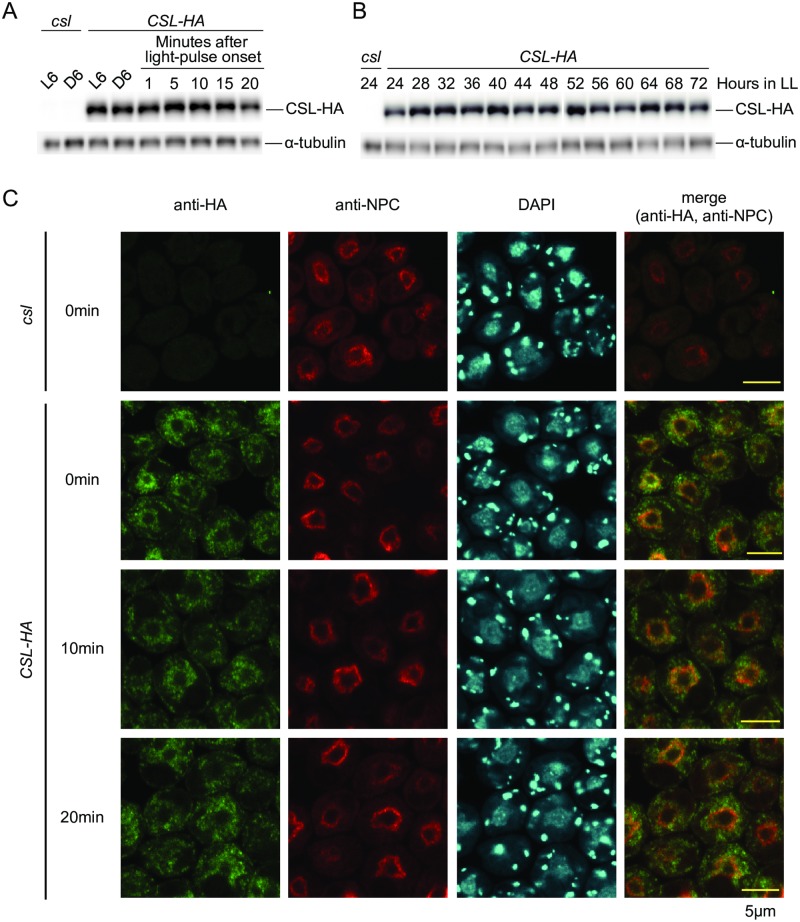
Expression analysis of CSL protein. **(A)** Western blot analysis of CSL-HA in LD and after light pulse. Cell were harvested from LD-entrained HS liquid cultures at midday and midnight. A red light pulse (2 μmol∙m^-2^∙s^-1^, 0.5 min) was given at midnight, and cells were harvested at times indicated in the graph. 2 μg of total protein was loaded in each lane. **(B)** Western blot analysis of CSL-HA in a circadian condition. Mid-log-phase (approximately 2 x 10^6^ cells/mL) HS liquid cultures at 24°C were transferred to 12 h darkness at 17°C to synchronize the circadian clock, and then released into a continuous light condition (2 μmol∙m^-2^∙s^-1^, white light, 17°C). 10 μg of total protein was loaded in each lane. Western blots for α-tubulin are shown as a reference protein (A,B). **(C)** Immunocytochemical staining of CSL-HA. Cells prepared as described in (A) were used. Cells were counterstained with NPC antibody and 4′,6-diamidino-2-phenylindole (DAPI). Photographs obtained under the same settings of microscope and detector are shown. *csl* mutant was used as negative controls (A,B,C).

To analyze expression of *CSL* gene product, we inserted a codon-adapted HA-epitope tag [21] at the end of the *CSL* coding sequence (CDS) of the gDNA fragment (Fragment 1; Fig 6A). This DNA fragment fully complemented the *csl* phenotype (S13 Fig), indicating that the tagged version of the CSL protein (CSL-HA) is functional. CSL-HA protein was expressed at almost the same level at midday and midnight in a 12-h light/dark cycle and after light pulse at midnight (Fig 8A). In addition, no obvious circadian rhythm was detected in the level of CSL-HA protein (Fig 8B). After immunocytochemical staining, CSL-HA signals were detected in perinuclear and cytoplasmic regions (Fig 8C), but no obvious changes in localization were detected after light pulse (Fig 8C). These results indicate that CSL protein is constitutively expressed and localized to the cytoplasm.

## Discussion

We previously demonstrated that the *ROC15* gene is necessary for normal phase-resetting in *Chlamydomonas* through analysis of an insertional *roc15* mutant expressing little or no *ROC15* mRNA [14,21]. However, it remained unclear whether the light-induced degradation of ROC15 was important for phase resetting or the presence of ROC15 was essential for it. In this study, we showed that phosphorylation/nuclear localization of ROC15 and circadian rhythm in DD are normal in *csl* mutant (Fig 4, S3 Fig). This indicates that ROC15 functions normally in the oscillatory mechanism of the circadian clock. It seems that the light response of ROC15 protein was specifically affected in *csl*. This supports our previous suggestion that the light-induced degradation event in ROC15 protein is important for the phase-resetting mechanism of the *Chlamydomonas* circadian clock.

Our results clearly showed that blue and red/violet signals are transmitted separately in the light-induced degradation of ROC15 protein (Fig 2). On the other hand, *b19* and *b20*, as well as *roc114-1* [21], showed their phenotypes in both blue and red light (S2 Fig). This result suggests that these signals are integrated into a common mechanism. It is not clear whether they are integrated at the point of ROC15 or upstream (e.g., the kinase for ROC15). Further analysis of *b19* and *b20* may reveal the integration point of these light signaling pathways.

In addition, we showed that *ROC15* mRNA downregulation by red light is independent of *CSL* (Fig 5). This finding suggests that there is another red light signaling pathway for mRNA regulation of *ROC15*. However, this is thought to be a supportive pathway in the phase resetting mechanism, because the red light response evident by the ROC15 protein level and phase resetting is largely abolished in *csl*, regardless of its normal mRNA response (Figs 2 and 3). ROC15 is a night phase expressed clock protein [21], and its mRNA level usually remains relatively high at the subjective dawn [14]. Thus, mRNA downregulation may be important to prevent re-accumulation of ROC15 protein and facilitate the night-to-day progression of the circadian clock.

CSL does not seem to be a light sensor because it does not possess known chromophore-binding domains; however, we do not exclude the possibility that its LRR may act as a novel chromophore binding site (Fig 7A). Although photoreceptor(s) in the CSL-related pathway remain unclear, it might be possible to speculate on the absorption spectrum from the equal-quantum action spectra in the WT and *csl* (Fig 2). S14 Fig shows a predicted contribution of the CSL pathway for ROC15 light response calculated by subtracting the sensitivity in the *csl* mutant from that in the WT. This perhaps reflects the *in vivo* action spectrum of CSL pathway. To our knowledge, there are no known photoreceptors in *Chlamydomonas* that show absorption spectra such as this. These spectral properties seem to be similar to some bilin-based photoreceptors, such as phytochromes and cyanobacteriochromes [40]. There are no obvious orthologs of them in the *Chlamydomonas* genome [26,41]; however, it is known that bilins (e.g. biliverdin [BV] and phycocyanobilin [PCB]) are synthesized in *Chlamydomonas* as retrograde signaling molecules from the chloroplast to the nucleus [42]. If an unknown bilin-based photoreceptor is involved in ROC15 light response, there is another possibility that CSL is related to bilin synthesis. However, this possibility is not probable given the fact that the *csl* phenotype was not rescued through the addition of bilins to the media (S15 Fig). In any case, CSL would be implicated in the red/violet signaling pathway. Further studies of CSL will shed light on a new light response mechanism of algae.

## Materials and methods

### *Chlamydomonas* strains and media

A reporter strain harboring ROC15-LUC gene in the nuclear genome was used as a WT strain [21]. In addition, CBR, *roc114-1*, and ROC15-HA strains [14,21] were also used. All strains are derivatives of CBR34 strain which shows a robust and stable bioluminescence rhythm [14]. Tris-acetate-phosphate (TAP) [43] and high-salt (HS) [44] media were used in this study.

### Transformation

Nuclear transformation of *Chlamydomonas* by electroporation was performed as described previously [21,45]. For generation of insertional mutants, 15–30 ng of transforming DNA was used per electroporation of 2.5 x 10^7^ cells.

### Bioluminescence monitoring

Luciferin was added to culture media at the final concentration of 100 or 200 μM. Cell cultures in white 96-well and black 24-well plates were measured using the integrated automatic bioluminescence monitoring apparatus [22] and the highly sensitive bioluminescence monitoring apparatus (CL24-W and CL24A-LIC; Churitsu Electric Corp., Nagoya, Japan), respectively. For monitoring during light phases, cells were kept in darkness for 210 s just before bioluminescence measurement at each time point to decrease the delayed light emission of chlorophyll. Parameters of circadian rhythms were calculated with the Rhythm-Analyzing Program [23].

### Light sources

We used fluorescence tubes (FLR 40S W/M; Panasonic) for white illumination and LEDs for violet (405 nm with a full-width at half maximum [FWHM] of 14 nm), blue (470 nm [FWHM of 27 nm]), and red (660 nm [FWHM of 24 nm]) illuminations (ISL-150×150-VV, ISL-150×150-BB, and ISL-150×150-RR, respectively; CCS, Kyoto, Japan). For screening, we used a larger size panel (30 × 60 cm) of red LED (660 nm [FWHM of 18 nm]) (LED Grow Light M; Home Grown, Yokohama, Japan). For the action spectra, we used a tunable monochromatic light source (PVL-3300; Asahi Spectra, Tokyo, Japan). Fluorescence rates were measured at the surface of plates or flasks using a light meter (Model LI-250; LI-COR, NE, USA) attached to a quantum probe (LI-190; LI-COR) or a multi-channel spectrometer (MC2100; Otsuka Electronics, Osaka, Japan). Spectrograms of light sources used in this study are shown in S16 Fig. We used an undetectable level of blue LED light as a safety light for harvesting the cells in darkness.

### Western blot analysis

Western blot analysis was performed as described previously [21]. We used rat monoclonal anti-HA antibody (1:10,000–1:50,000; clone 3F10, Roche), mouse monoclonal anti-HA antibody (1:1,000; COVANCE, clone 16B12), and mouse monoclonal anti-FLAG antibody (1:1,000; Sigma, clone M2) as the primary antibody. Horseradish peroxidase (HRP) conjugated antibodies were used as the secondary antibody.

### Immunocytochemistry

Immunocytochemistry was performed as described previously [21]. For detection of HA-tagged proteins, we used rat monoclonal anti-HA antibody (clone 3F10; 1:1,000; Roche) and Alexa488-conjugated goat anti-rat IgG (Thermo Fisher Scientific) as the primary and secondary antibodies, respectively. Fluorescence was observed with a laser-scanning confocal fluorescence microscope (FV10i-DOC, Olympus).

### Genetic analyses for identification of disrupted gene

Genetic crosses, Southern blot analysis, and TAIL-PCR were performed as described previously [14].

### RT-PCR and RT-qPCR

Total RNA was extracted using TRIzol reagent (ThermoFisher Scientific). RNA concentrations were determined using a spectrophotometer (UVmini-1240; SHIMADZU). For RT-PCR, reverse transcription was performed using SuperScript II (ThermoFisher Scientific) or ReverTra Ace qPCR RT Master Mix (TOYOBO), and following PCR amplification was performed using KOD FX neo (TOYOBO). For RT-qPCR, one microgram of total RNA was used in each reverse transcription reaction using ReverTra Ace qPCR RT Master Mix. Following qPCR was performed using KOD FX neo supplemented with SYBR^®^ Green I Nucleic Acid Gel Stain (TaKaRa) and ROX dye (ThermoFisher Scientific) at final concentrations of 0.05x (200,000-fold dilution) and 0.5 μM, respectively. We used StepOnePlus (ThermoFisher Scientific) for qPCR at the following cycling conditions with standard ramp speed: 2 min at 94°C, 40 x (10 sec at 98°C, 1 min at 68°C). Relative transcript abundances were determined by the relative standard curve method of StepOnePlus using *RCK1* as a reference gene for normalization [46]. Quantification standards for each target cDNA were obtained using band-purified PCR products as templates. In order to avoid amplifying contaminating genomic DNA, we designed all qPCR primers to anneal across a splicing junction of transcript, and to contain at least one intron between the primer pairs. Primers used for this study are provided in S1 Table. Target specificity was confirmed by melt curve method for all samples and by gel electrophoresis and sequencing for some representative samples.

### Plasmid construction

Plasmids for complementation of *csl* by gDNA fragments were generated as follows: The paromomycin resistance cassette of pAC103PL [47] was replaced with the spectinomycin resistance cassette from pALM32 [48] by ligating the 3.3-kb *Not*I-*Bam*HI fragment of pAC103PL and the 2.2-kb *Not*I-*Bam*HI fragment of pALM32. The resulting plasmid termed pLaadA has a multicloning site from pAC103PL upstream of the spectinomycin resistance cassette. A bacterial artificial chromosome (BAC) clone (CRB1-063O12) carrying a *csl* locus was obtained from the genomic library described in the Kyoto *Chlamydomonas* Genome Database (KCGD; http://www.molecule.lif.kyoto-u.ac.jp/kcgd.html). Furthermore, 7.4-kb *Avr*II/*Ase*I (Fragment 1), 10.8-kb *Avr*II/*Ase*I (Fragment 2), and 17.4-kb *Bsp*EI/*Ase*I (Fragment 3) fragments were cloned into the multicloning site of pLaadA. For HA- and FLAG-tagged construct, the stop codon of *CSL* of Fragment 1 was replaced with an *Fse*I restriction site, and codon adapted HA [21] or FLAG (S10 Fig) tag sequences was inserted. To remove vector backbone sequences, we digested the plasmids with *Pac*I, and the fragments carrying the gDNA linked to the spectinomycin resistance cassette were used for *csl* mutant transformation.

A plasmid for complementation by *CSL* CDS was generated as follows: The *CSL* CDS was amplified by *Dra*I and *Eco*RI site attached primers just upstream and downstream of the CDS, respectively. The *Dra*I-*Eco*RI fragment was cloned into the *Nae*I/*Eco*RI digested pAR [47] to generate pAR/CSL. The *Pac*I fragment containing the *CSL* expression cassette was co-transformed with the spectinomycin resistant cassette [48].

## Supporting information

S1 Fig Abnormal diurnal rhythm of ROC15-LUC bioluminescence in *roc114-1*. A *roc114-1* mutant strain with a ROC15-LUC reporter was used. Cultures of the strain were prepared as described in Fig 1 and their bioluminescence was monitored.
(TIF)

S2 Fig Acute response of ROC15-LUC to red and blue light pulses. Asynchronous TAP liquid cultures of *b16*, *b19*, and *b20* in black 24-well plates were subjected to darkness for 6 h for accumulation of ROC15-LUC, and then a 5 min light pulse (Red: 2 μmol∙m^-2^∙s^-1^; Blue: 20 μmol∙m^-2^∙s^-1^) was administered by blue and red LED panels (arrows).
(TIF)

S3 Fig Circadian rhythm of *b16* mutant. To replace the ROC15-LUC reporter gene in *b16* with the chloroplast reporter gene of CBR, the *b16* strain (mating type minus [mt^-^]) was genetically crossed with the CBR strain (mt^+^). All 96 progenies showed bioluminescence due to uniparental inheritance of chloroplast DNA. We omitted paromomycin-resistance progenies because they possess ROC15-LUC introduced into the genome with the paromomycin-resistance *APHVIII* gene [21]. Genotype was confirmed definitively by genomic PCR. **(A)** Parameters of the bioluminescence rhythm of progeny. 5-day old spot cultures of progenies on HS agar in white 96-well plates were subjected to 12 h dark/12 h light to synchronize the circadian clock, and then the bioluminescence rhythm was monitored in DD. A scatter plot of period length and phase angle of circadian bioluminescence rhythms of hygromycin-resistant (Hyg^R^) and sensitive (Hyg^S^) progenies is shown. The resistance is genetically linked to *b16* (see S5 Fig). **(B)** Representative trace of a progeny of WT and *b16*. Genotypes of some progenies were confirmed by genomic PCR, and representative WT and *b16* progenies were subjected to a repeated rhythm assay. Data are the mean ± SD of relative bioluminescence (the average of the third peak was set to 100) from 10 independent cultures of the progenies.
(TIF)

S4 Fig Bioluminescence trace of the ROC15-LUC reporter strain in the same plate as the phase shifting experiment of Fig 3A. Cells were treated as described in Fig 3A. Data before and after the light pulse (arrows) are shown. The bioluminescence level just before light pulse was set to 100. Each point represents the mean ± SD of 10 independent cultures.
(TIF)

S5 Fig Identification of the disrupted gene in *b16*. **(A)** Co-segregation of the *b16* and hygromycin-resistance phenotypes. The *b16* mutant was backcrossed to a WT (ROC15-LUC) strain, and then the bioluminescence response to red light and hygromycin-resistance of progenies were tested. The left panel shows ROC15-LUC bioluminescence of progenies monitored under the same conditions as Fig 1. Data around the light onset of first DL cycle are shown. The bioluminescence level just before light pulse was set to 100. The right panel is a histogram representing the distribution of bioluminescence levels of progenies after light on (relative to the dark phase). **(B)** Southern blot analysis of *b16* gDNA. Genomic DNA from *b16* cells was digested by restriction enzymes without recognition (restriction) sites in the *aph7”* marker gene. The three lanes are distant lanes within the same gel. **(C, D)** TAIL-PCR products (C) and PCR product of specific primers overlapping the insertion site (D). These products were separated on an agarose gel and stained with ethidium bromide. The TAIL-PCR product used for sequencing analysis is indicated by an arrowhead.
(TIF)

S6 Fig RT-PCR analysis of Cre02.g092150 in WT, *b16* mutant, and complemented transformants (Comp.1, Comp.2). **(A)** Schematic representation of primer locations. **(B)** RT-PCR result by using primers bracketing the insertion site (A, Red arrows). The solid and open triangle indicate RT-PCR products from a WT and mutant transcript, respectively. The mutant transcript was longer than that of WT probably due to insertion of Hyg maker. **(C)** Quantification of transcripts. The total amount of Cre02.g092150 transcripts was determined by RT-qPCR by using primers indicated in A as blue arrows. The transcript abundances relative to *RCK1* were further normalized by dividing by the WT level for easy comparison.
(TIF)

S7 Fig Analysis of upstream genes of Cre02.g092150. **(A)** Schematic representation of the *b16* locus (shown in reverse orientation of chromosome 2 sequence). Gene models and encoded protein motifs are shown: the FK506-binding-protein-type peptidyl-prolyl *cis*-*trans* isomerase (FKBP_C), tetratricopeptide repeat (TPR), pyrroloquinoline quinone (PQQ), and leucine-rich repeat (LRR) domains. Fourteen forward primers (1–14) and one reverse primer (15) for RT-PCR analysis are represented by red and blue arrows, respectively. The bars at the bottom indicate genomic DNA fragments for complementation analysis. **(B)** RT-PCR analysis of the *b16* locus. RT-PCR products (25 cycles) were separated on an agarose gel and stained with ethidium bromide. **(C)** Complementation of the *b16* phenotype by gDNA fragments. Data are collected and shown as Fig 6B, 6C and 6D.
(TIF)

S8 Fig Complementation of the violet light response of ROC15-LUC by Cre02.g092150. Asynchronous TAP liquid cultures of WT, *b16*, and the complemented strains in black 24-well plates were subjected to darkness for 3.5 h for accumulation of ROC15-LUC, and then a 0.5 min violet light pulse of indicated intensities was administered by a violet LED panel (arrows). The graphs show representative bioluminescence traces. The bioluminescence level just before light pulse was set to 100.
(TIF)

S9 Fig Complementation of the re-entrainment phenotype by Cre02.g092150. The *b16* mutant harboring the chloroplast bioluminescence reporter (S3 Fig) was transformed with the Fragment 1. Re-entrainment of transformants to the 5 h advanced light cycles was observed in experimental conditions essentially the same as in Fig 3C. **(A)** Bioluminescence traces. A representative trace of a complemented transformant is shown. WT and *b16* traces are shown for comparison. Bioluminescence levels were detrended by dividing by the 24 h moving average. **(B)** Histograms representing the distribution of the amount of phase shift of all transformants (phase difference between transformants and a dark control of *b16*). Asterisk indicates complemented transformants (the phase shift > 4.5 h). Numbers of complemented transformants and the rate of complementation are indicated in the graphs. **(C)** The amount of phase shift to red, blue, and violet light cycles of the complemented transformants (Comp.1 and Comp2). Graphs show the mean ± SD of 10 independent cultures of the phase difference (h) between light-entrained and dark control samples. * *P* < 0.01, ** *P* < 0.001 (Student’s *t*-test).
(TIF)

S10 Fig Generation of Cre02.g092150-FLAG strain. **(A)** Nucleotide and amino acid sequences of a codon-adapted FLAG tag for *C*. *reinhardtii* nuclear genome. **(B)** A schematic representation of the FLAG-tagged Cre02.g092150 gene. Black and gray boxes represent the CDS and untranslated regions (UTRs), respectively. **(C)** Complementation of the *b16* phenotype. Asynchronous cultures of transformants of *b16* mutant with Cre02.g092150-FLAG were subjected to darkness for 3 h for accumulation of ROC15-LUC, and then a 0.5 min red light pulse (660 nm, 2 μmol∙m^-2^∙s^-1^) was administered (arrow). The left graph shows representative bioluminescence traces, and the right panel is a histogram representing the distribution of bioluminescence level of all transformants after light pulse (relative to the pre-light pulse level). Asterisk represents complemented transformants. Numbers of complemented transformants and the complementation rate are indicated in the graph. **(D)** Western blot analysis of Cre02.g092150-FLAG. Cells of asynchronous cultures of complemented strains were harvested and subjected to Western blot analysis with an anti-FLAG antibody. A protein sample of *b16* mutant was loaded as a negative control.
(TIF)

S11 Fig Complementation of the ROC15 phosphorylation phenotype in *b16*. The Cre02.g092150-FLAG strain was crossed with the ROC15-HA strain [21], and progenies harboring the ROC15-HA and both the *b16* mutation and Cre02.g092150-FLAG transgenes (Cre02.g092150-FLAG/*b16*) were selected by spot tests for antibiotic resistance and genotyping by genomic PCR. Cell cultures were prepared as described in Fig 4B, and exposed to a 0.5 min pulse of red light (660 nm, 2 μmol∙m^-2^∙s^-1^) or violet light (405 nm, 2 μmol∙m^-2^∙s^-1^) at midnight. Total protein extracts were subjected to Western blot analysis using anti-HA antibody.
(TIF)

S12 Fig Red light response of *ROC40* mRNA in a complemented strain of *b16*. Samples of red light exposed cells were prepared as described in Fig 5. RT-qPCR results of *ROC40* of the WT, *b16*, and the complemented strain (Comp1; Fig 6B, S6 Fig) are shown. The transcript abundances relative to *RCK1* were further normalized by dividing by the dark control level of the WT for easy comparison.
(TIF)

S13 Fig Generation of the CSL-HA strain. **(A)** Schematic representation of the *CSL-HA* gene. Black and gray boxes represent the CDS and UTRs, respectively. **(B)** Complementation of the *csl* phenotype by *CSL-HA*. *csl* mutant was transformed with *CSL-HA* gene. Data are collected and shown as Fig 6B, 6C and 6D. **(C)** Western blot analysis of CSL-HA. Cells of asynchronous TAP liquid cultures of the complemented strains were harvested and subjected to Western blot analysis with an anti-HA antibody. A protein sample of *csl* mutant was loaded as a negative control. **(D)** Light dose dependency of the light response of ROC15-LUC in CSL-HA strain. Asynchronous cultures of the CSL-HA strains were subjected to darkness for 3 h for accumulation of ROC15-LUC, and then 2 min red light pulses of indicated intensities were administered. Data indicated are relative bioluminescence levels of CSL-HA-expressed strains against that of the *csl* mutant 30 min after light pulse onset.
(TIF)

S14 Fig A predicted *in vivo* action spectrum of the CSL light signaling pathway. The sensitivity of ROC15 light response remaining in *csl* was subtracted from that of WT. Negative values were regarded as 0.
(TIF)

S15 Fig Effect of bilins in the ROC15-LUC response to red light. Asynchronous TAP cultures of WT and *csl* in black 24-well plates were subjected to darkness for 3 h for accumulation of ROC15-LUC, and then a red light pulse (2 μmol∙m^-2^∙s^-1^) were administered for 0.5 min (arrows). Billins (Frontier Scientific, UT, USA) were added to the cultures just before dark adaptation at the final concentrations indicated in the graphs. Each trace is the mean of bioluminescence from two independent cultures. The bioluminescence level just before light pulse was set to 100.
(TIF)

S16 Fig Spectrograms of light sources used in this study. **(A)** Spectrograms of monochromatic light emitted by the tunable monochromatic light source PVL-3300 (Asahi Spectra, Tokyo, Japan). **(B)** Emission spectra of the violet, blue, and red LED (ISL-150×150-VV, ISL-150×150-BB, and ISL-150×150-RR, respectively; CCS, Kyoto, Japan).
(TIF)

S1 Table List of primers. (XLSX)
