# Supplementary figures and images for: CSL encodes a leucine-rich-repeat protein implicated in red/violet light signaling to the circadian clock in Chlamydomonas
Source: PLoS Genet. 2017 Mar 23;13(3):e1006645. doi: 10.1371/journal.pgen.1006645 (PMC5363811; doi:10.1371/journal.pgen.1006645)

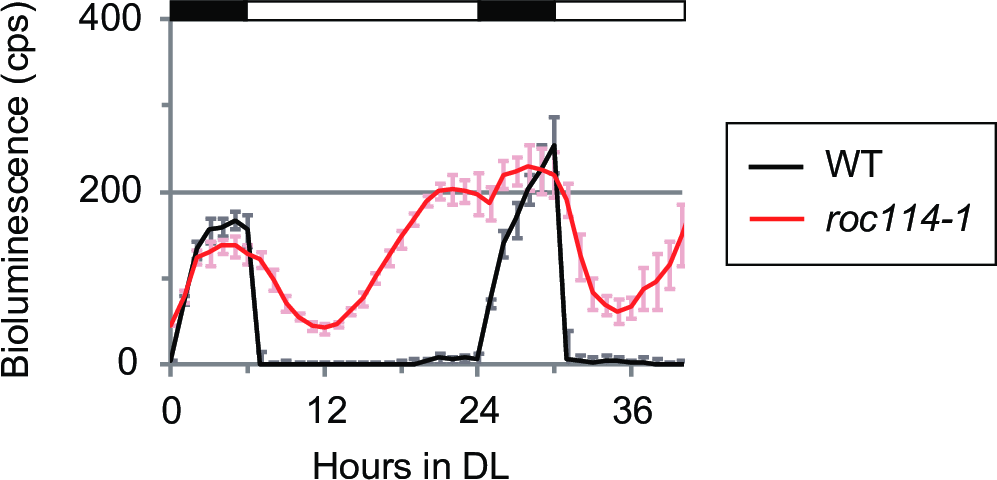

Supplement: S1 Fig — A roc114-1 mutant strain with a ROC15-LUC reporter was used. Cultures of the strain were prepared as described in Fig 1 and their bioluminescence was monitored. (TIF) [file pgen.1006645.s001.tif]

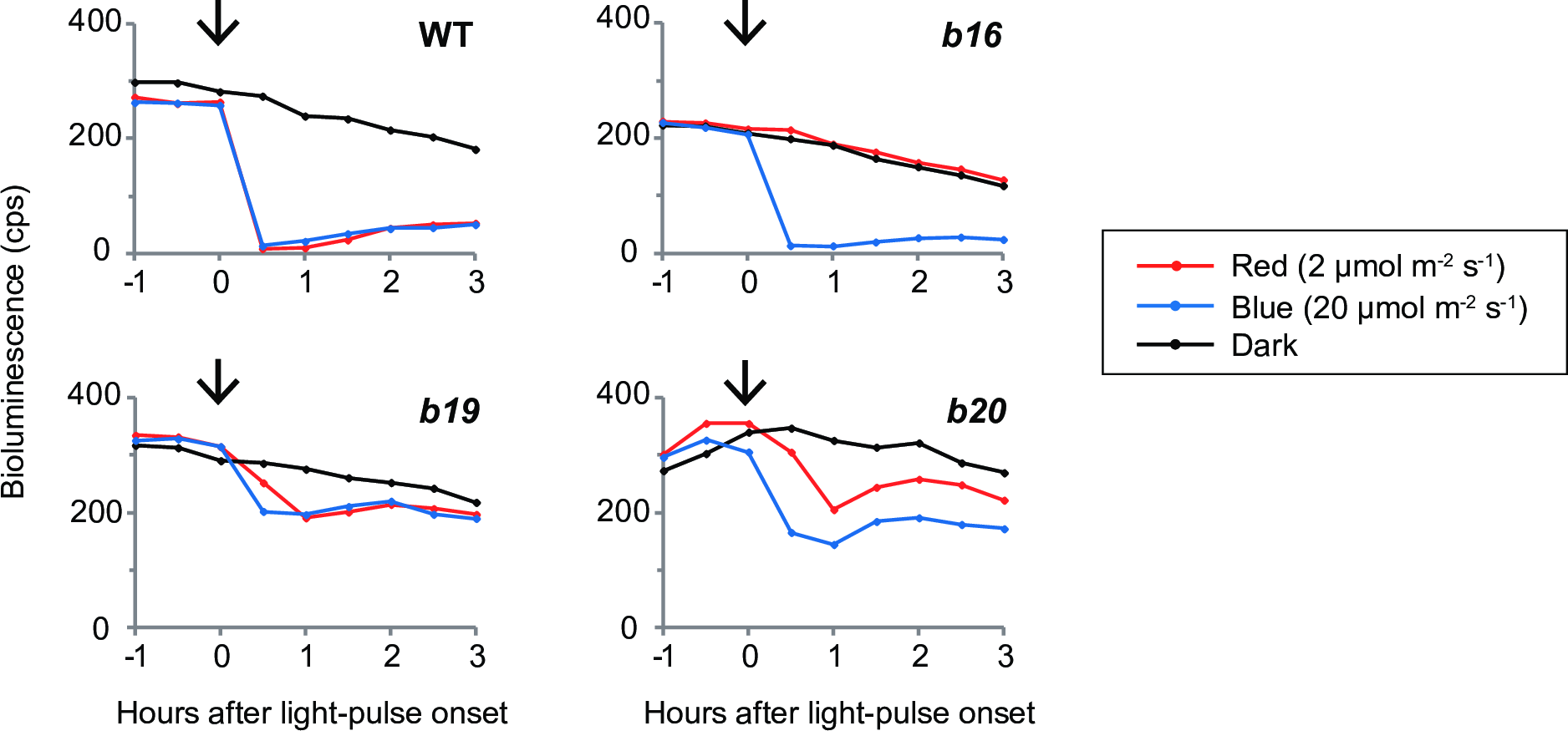

Supplement: S2 Fig — Asynchronous TAP liquid cultures of b16, b19, and b20 in black 24-well plates were subjected to darkness for 6 h for accumulation of ROC15-LUC, and then a 5 min light pulse (Red: 2 μmol∙m-2∙s-1; Blue: 20 μmol∙m-2∙s-1) was administered by blue and red LED panels (arrows). (TIF) [file pgen.1006645.s002.tif]

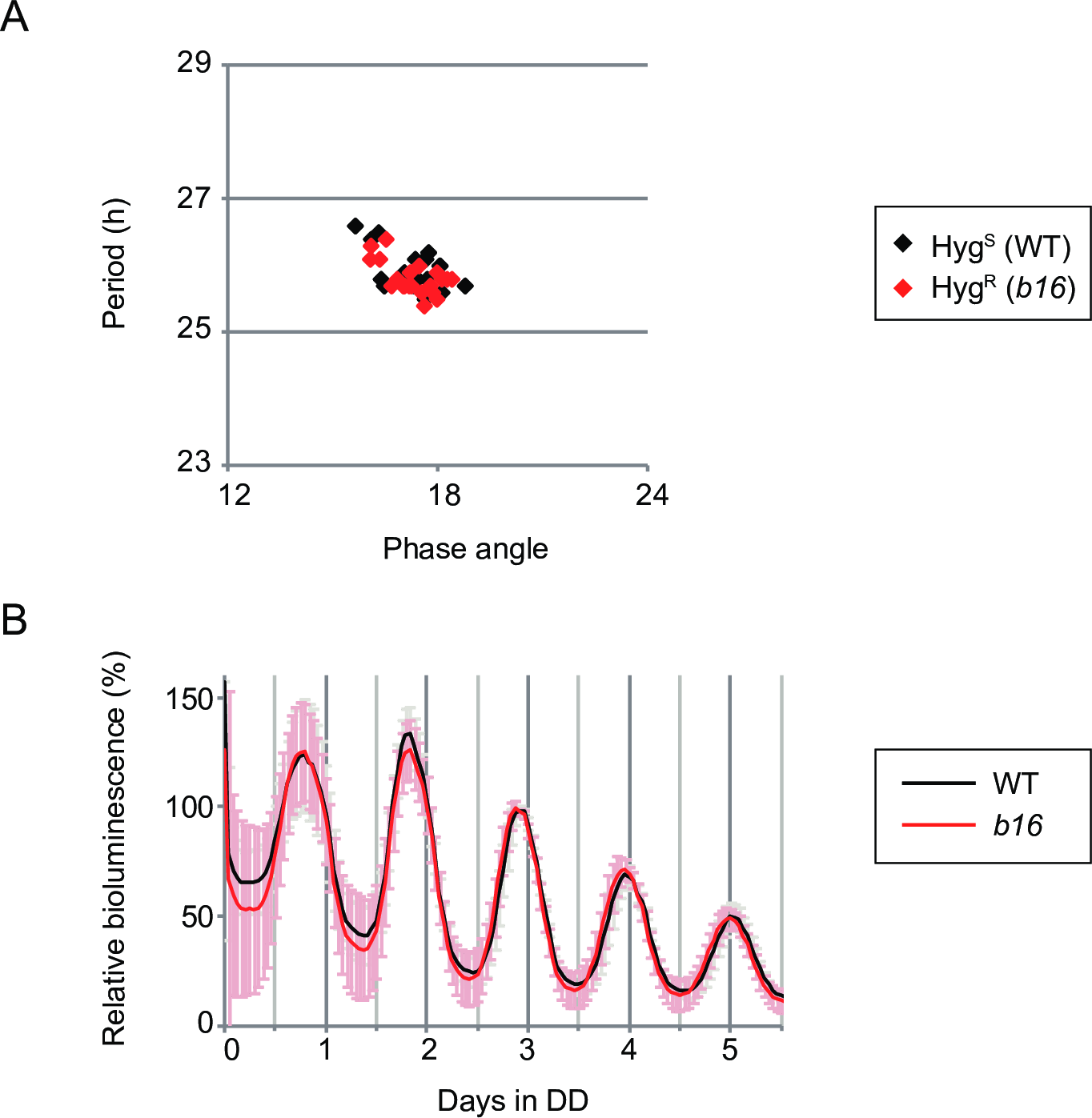

Supplement: S3 Fig — To replace the ROC15-LUC reporter gene in b16 with the chloroplast reporter gene of CBR, the b16 strain (mating type minus [mt-]) was genetically crossed with the CBR strain (mt+). All 96 progenies showed bioluminescence due to uniparental inheritance of chloroplast DNA. We omitted paromomycin-resistance progenies because they possess ROC15-LUC introduced into the genome with the paromomycin-resistance APHVIII gene [21]. Genotype was confirmed definitively by genomic PCR. (A) Parameters of the bioluminescence rhythm of progeny. 5-day old spot cultures of progenies on HS agar in white 96-well plates were subjected to 12 h dark/12 h light to synchronize the circadian clock, and then the bioluminescence rhythm was monitored in DD. A scatter plot of period length and phase angle of circadian bioluminescence rhythms of hygromycin-resistant (HygR) and sensitive (HygS) progenies is shown. The resistance is genetically linked to b16 (see S5 Fig). (B) Representative trace of a progeny of WT and b16. Genotypes of some progenies were confirmed by genomic PCR, and representative WT and b16 progenies were subjected to a repeated rhythm assay. Data are the mean ± SD of relative bioluminescence (the average of the third peak was set to 100) from 10 independent cultures of the progenies. (TIF) [file pgen.1006645.s003.tif]

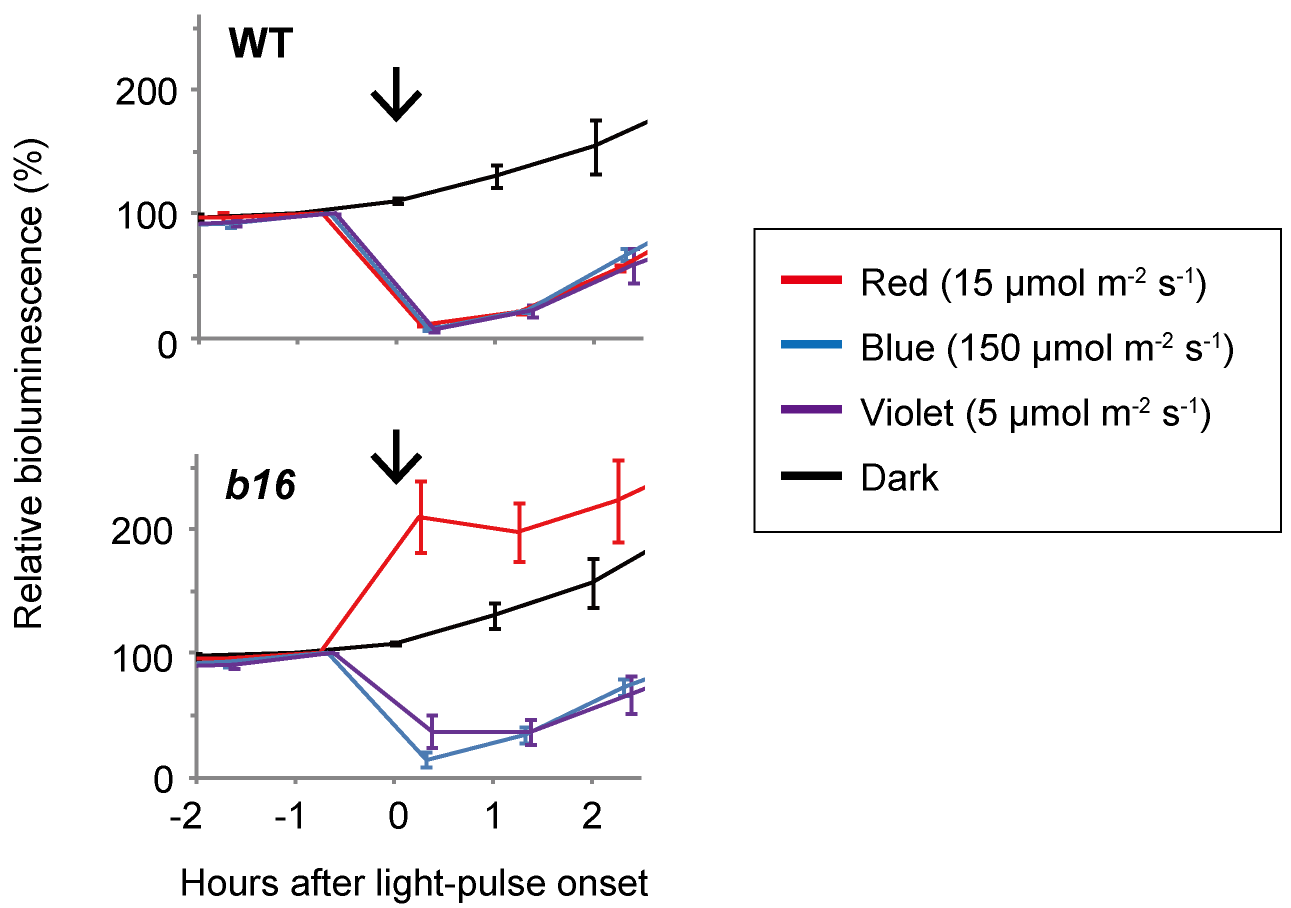

Supplement: S4 Fig — Cells were treated as described in Fig 3A. Data before and after the light pulse (arrows) are shown. The bioluminescence level just before light pulse was set to 100. Each point represents the mean ± SD of 10 independent cultures. (TIF) [file pgen.1006645.s004.tif]

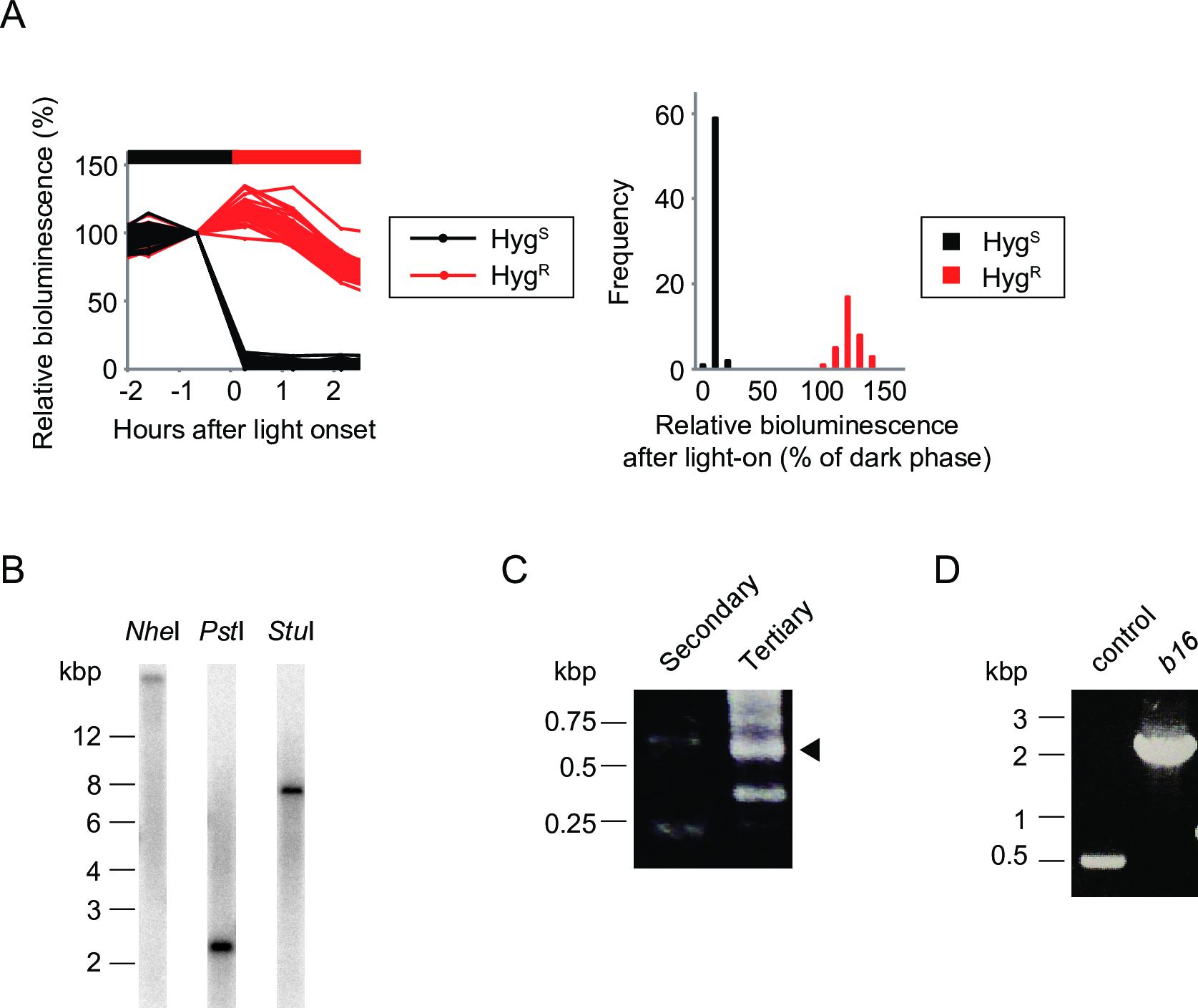

Supplement: S5 Fig — (A) Co-segregation of the b16 and hygromycin-resistance phenotypes. The b16 mutant was backcrossed to a WT (ROC15-LUC) strain, and then the bioluminescence response to red light and hygromycin-resistance of progenies were tested. The left panel shows ROC15-LUC bioluminescence of progenies monitored under the same conditions as Fig 1. Data around the light onset of first DL cycle are shown. The bioluminescence level just before light pulse was set to 100. The right panel is a histogram representing the distribution of bioluminescence levels of progenies after light on (relative to the dark phase). (B) Southern blot analysis of b16 gDNA. Genomic DNA from b16 cells was digested by restriction enzymes without recognition (restriction) sites in the aph7” marker gene. The three lanes are distant lanes within the same gel. (C, D) TAIL-PCR products (C) and PCR product of specific primers overlapping the insertion site (D). These products were separated on an agarose gel and stained with ethidium bromide. The TAIL-PCR product used for sequencing analysis is indicated by an arrowhead. (TIF) [file pgen.1006645.s005.tif]

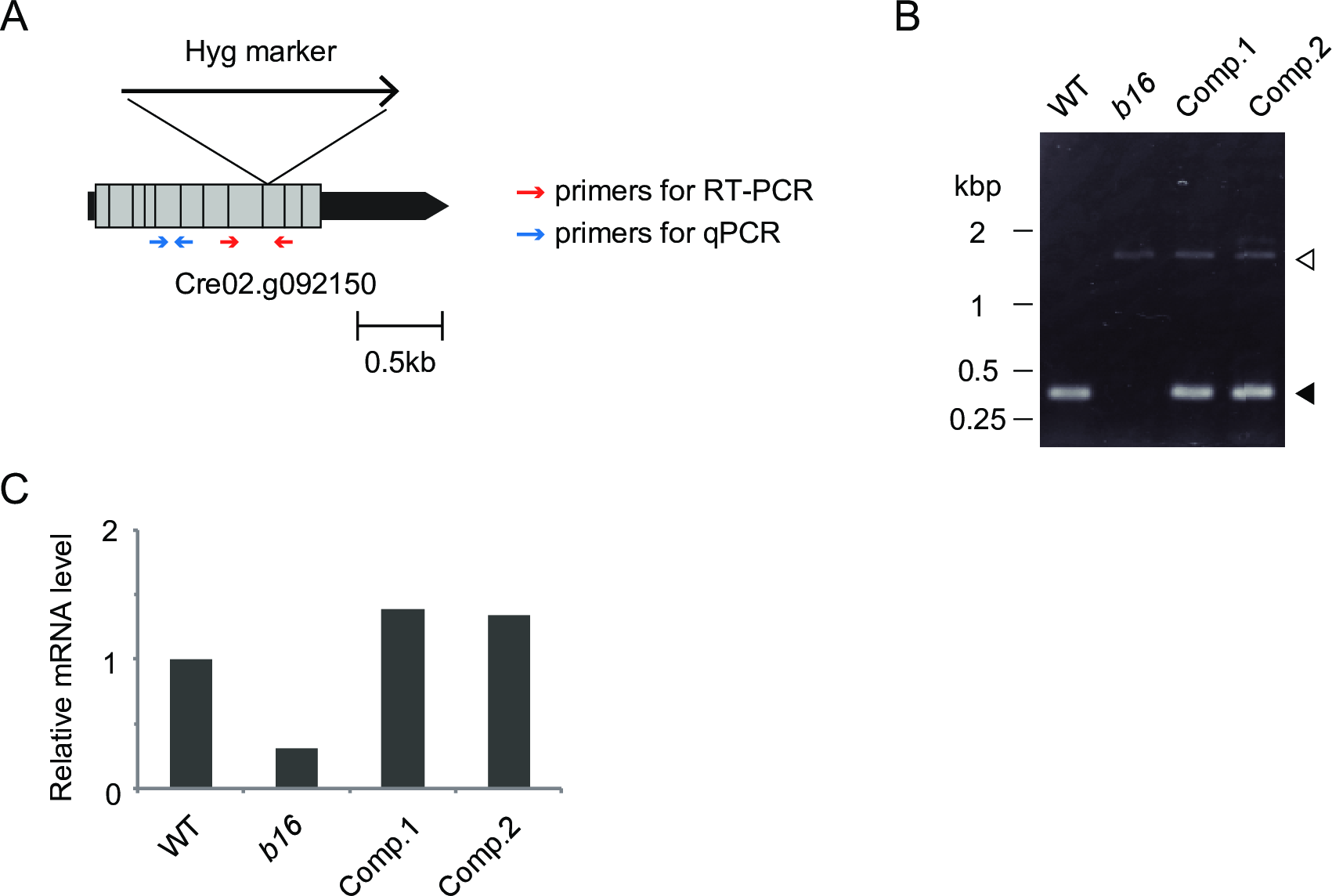

Supplement: S6 Fig — (A) Schematic representation of primer locations. (B) RT-PCR result by using primers bracketing the insertion site (A, Red arrows). The solid and open triangle indicate RT-PCR products from a WT and mutant transcript, respectively. The mutant transcript was longer than that of WT probably due to insertion of Hyg maker. (C) Quantification of transcripts. The total amount of Cre02.g092150 transcripts was determined by RT-qPCR by using primers indicated in A as blue arrows. The transcript abundances relative to RCK1 were further normalized by dividing by the WT level for easy comparison. (TIF) [file pgen.1006645.s006.tif]

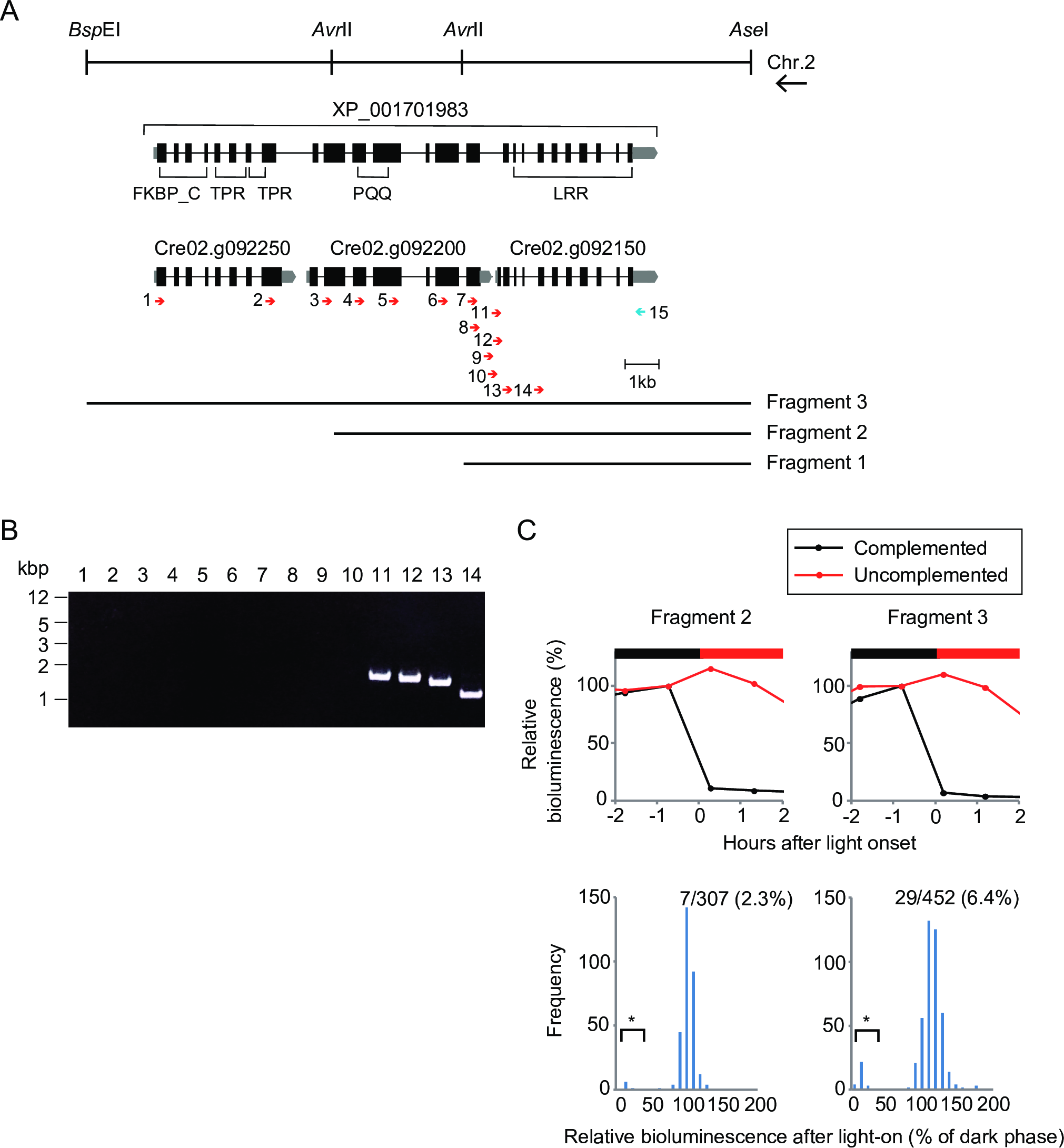

Supplement: S7 Fig — (A) Schematic representation of the b16 locus (shown in reverse orientation of chromosome 2 sequence). Gene models and encoded protein motifs are shown: the FK506-binding-protein-type peptidyl-prolyl cis-trans isomerase (FKBP_C), tetratricopeptide repeat (TPR), pyrroloquinoline quinone (PQQ), and leucine-rich repeat (LRR) domains. Fourteen forward primers (1–14) and one reverse primer (15) for RT-PCR analysis are represented by red and blue arrows, respectively. The bars at the bottom indicate genomic DNA fragments for complementation analysis. (B) RT-PCR analysis of the b16 locus. RT-PCR products (25 cycles) were separated on an agarose gel and stained with ethidium bromide. (C) Complementation of the b16 phenotype by gDNA fragments. Data are collected and shown as Fig 6B, 6C and 6D. (TIF) [file pgen.1006645.s007.tif]

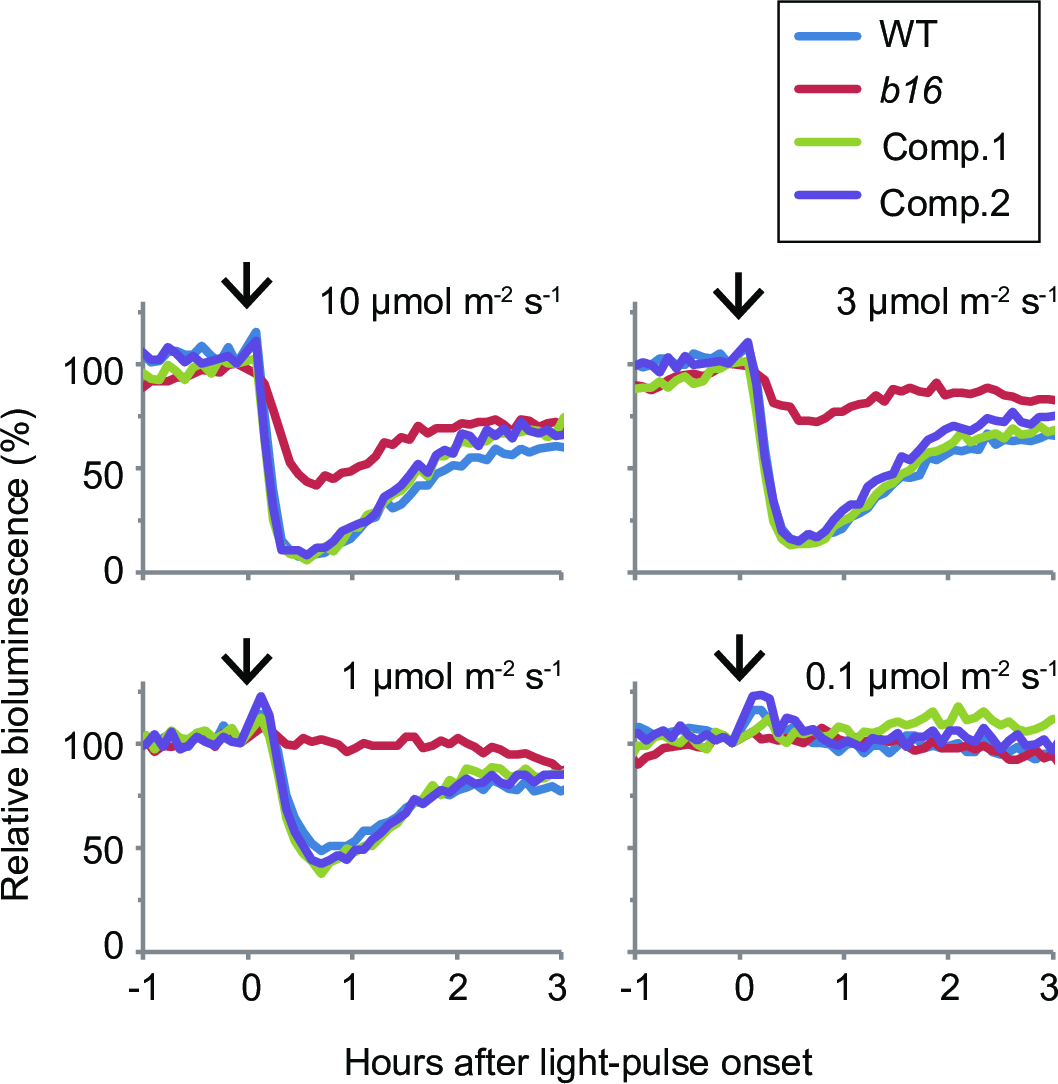

Supplement: S8 Fig — Asynchronous TAP liquid cultures of WT, b16, and the complemented strains in black 24-well plates were subjected to darkness for 3.5 h for accumulation of ROC15-LUC, and then a 0.5 min violet light pulse of indicated intensities was administered by a violet LED panel (arrows). The graphs show representative bioluminescence traces. The bioluminescence level just before light pulse was set to 100. (TIF) [file pgen.1006645.s008.tif]

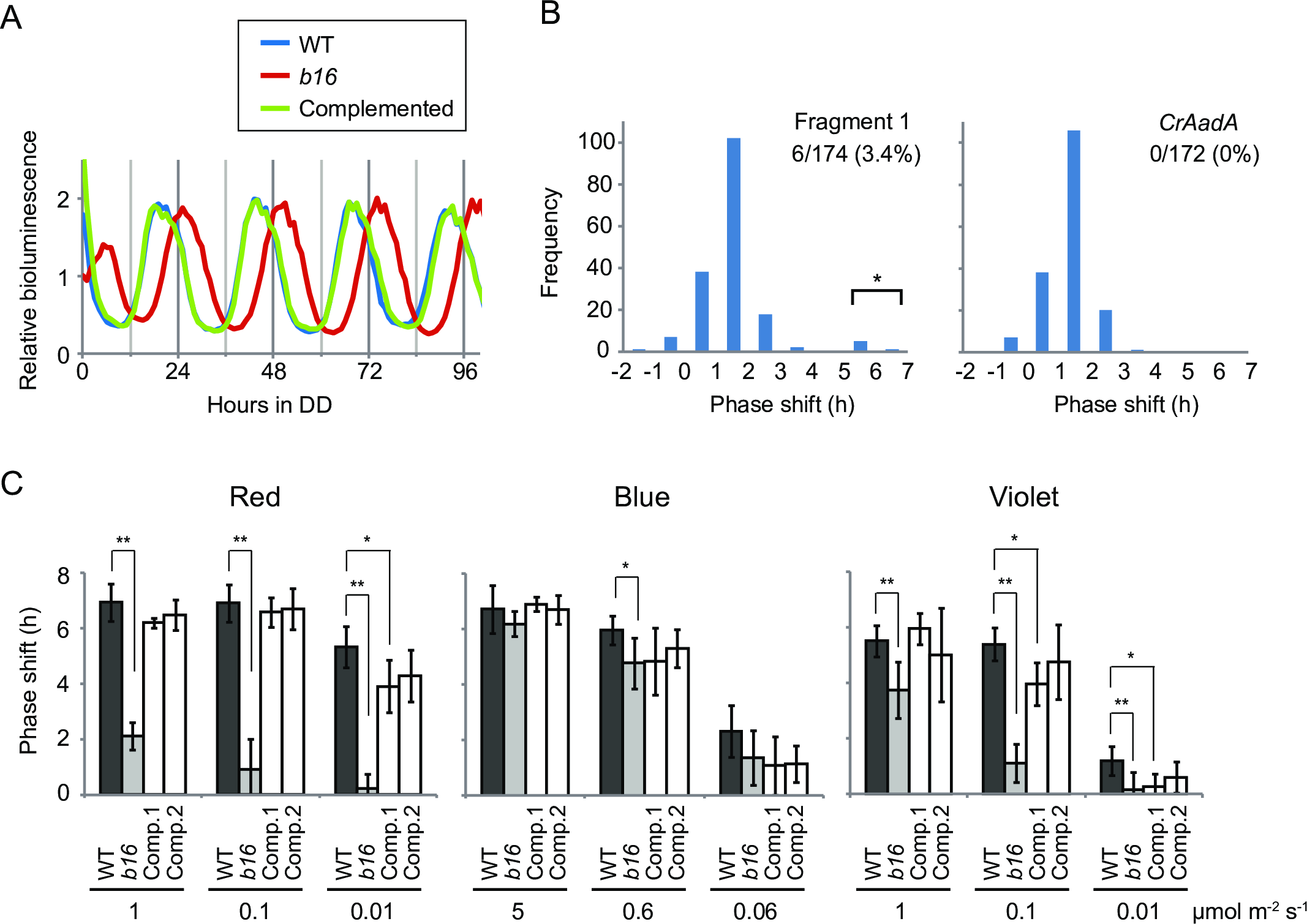

Supplement: S9 Fig — The b16 mutant harboring the chloroplast bioluminescence reporter (S3 Fig) was transformed with the Fragment 1. Re-entrainment of transformants to the 5 h advanced light cycles was observed in experimental conditions essentially the same as in Fig 3C. (A) Bioluminescence traces. A representative trace of a complemented transformant is shown. WT and b16 traces are shown for comparison. Bioluminescence levels were detrended by dividing by the 24 h moving average. (B) Histograms representing the distribution of the amount of phase shift of all transformants (phase difference between transformants and a dark control of b16). Asterisk indicates complemented transformants (the phase shift > 4.5 h). Numbers of complemented transformants and the rate of complementation are indicated in the graphs. (C) The amount of phase shift to red, blue, and violet light cycles of the complemented transformants (Comp.1 and Comp2). Graphs show the mean ± SD of 10 independent cultures of the phase difference (h) between light-entrained and dark control samples. * P < 0.01, ** P < 0.001 (Student’s t-test). (TIF) [file pgen.1006645.s009.tif]

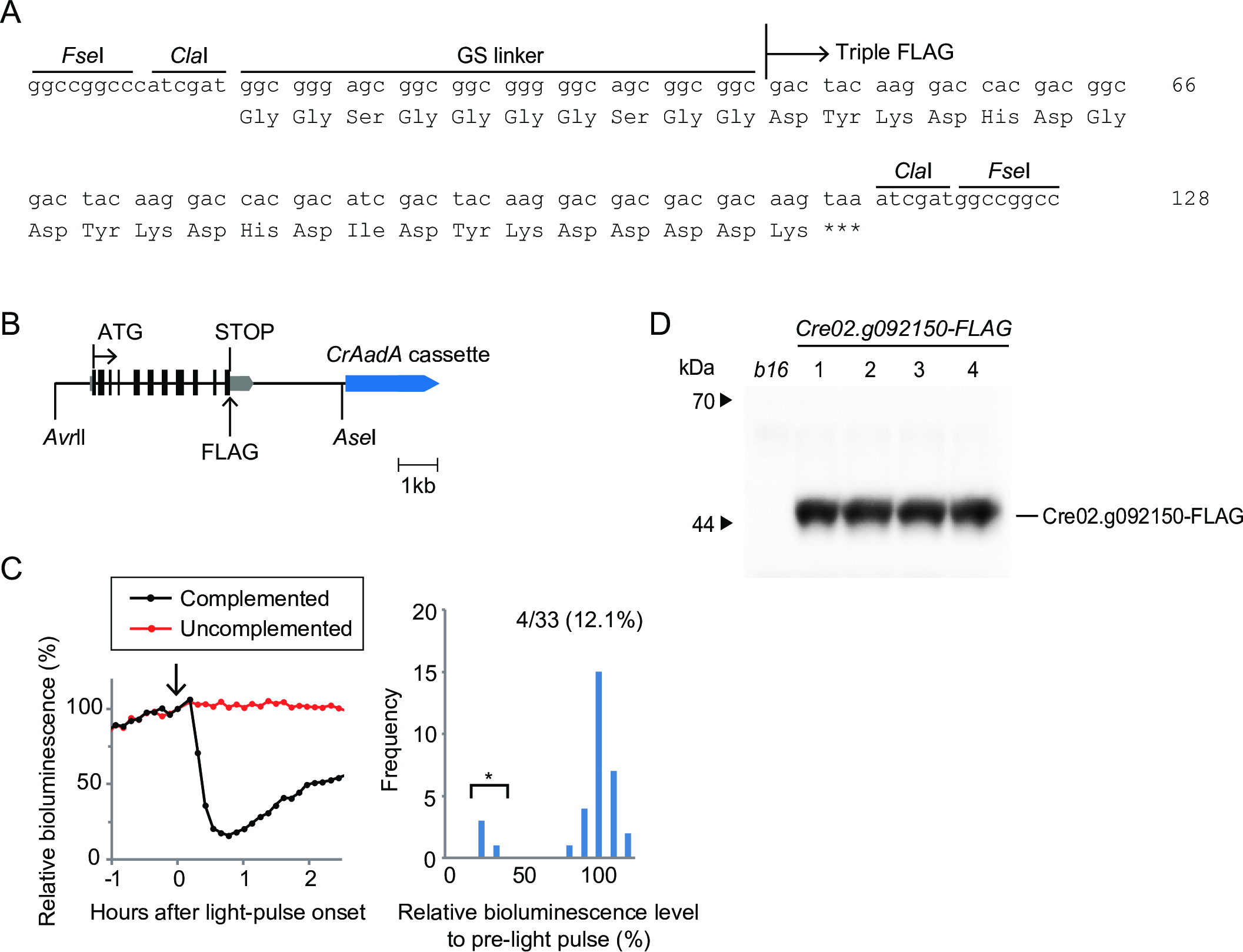

Supplement: S10 Fig — (A) Nucleotide and amino acid sequences of a codon-adapted FLAG tag for C. reinhardtii nuclear genome. (B) A schematic representation of the FLAG-tagged Cre02.g092150 gene. Black and gray boxes represent the CDS and untranslated regions (UTRs), respectively. (C) Complementation of the b16 phenotype. Asynchronous cultures of transformants of b16 mutant with Cre02.g092150-FLAG were subjected to darkness for 3 h for accumulation of ROC15-LUC, and then a 0.5 min red light pulse (660 nm, 2 μmol∙m-2∙s-1) was administered (arrow). The left graph shows representative bioluminescence traces, and the right panel is a histogram representing the distribution of bioluminescence level of all transformants after light pulse (relative to the pre-light pulse level). Asterisk represents complemented transformants. Numbers of complemented transformants and the complementation rate are indicated in the graph. (D) Western blot analysis of Cre02.g092150-FLAG. Cells of asynchronous cultures of complemented strains were harvested and subjected to Western blot analysis with an anti-FLAG antibody. A protein sample of b16 mutant was loaded as a negative control. (TIF) [file pgen.1006645.s010.tif]

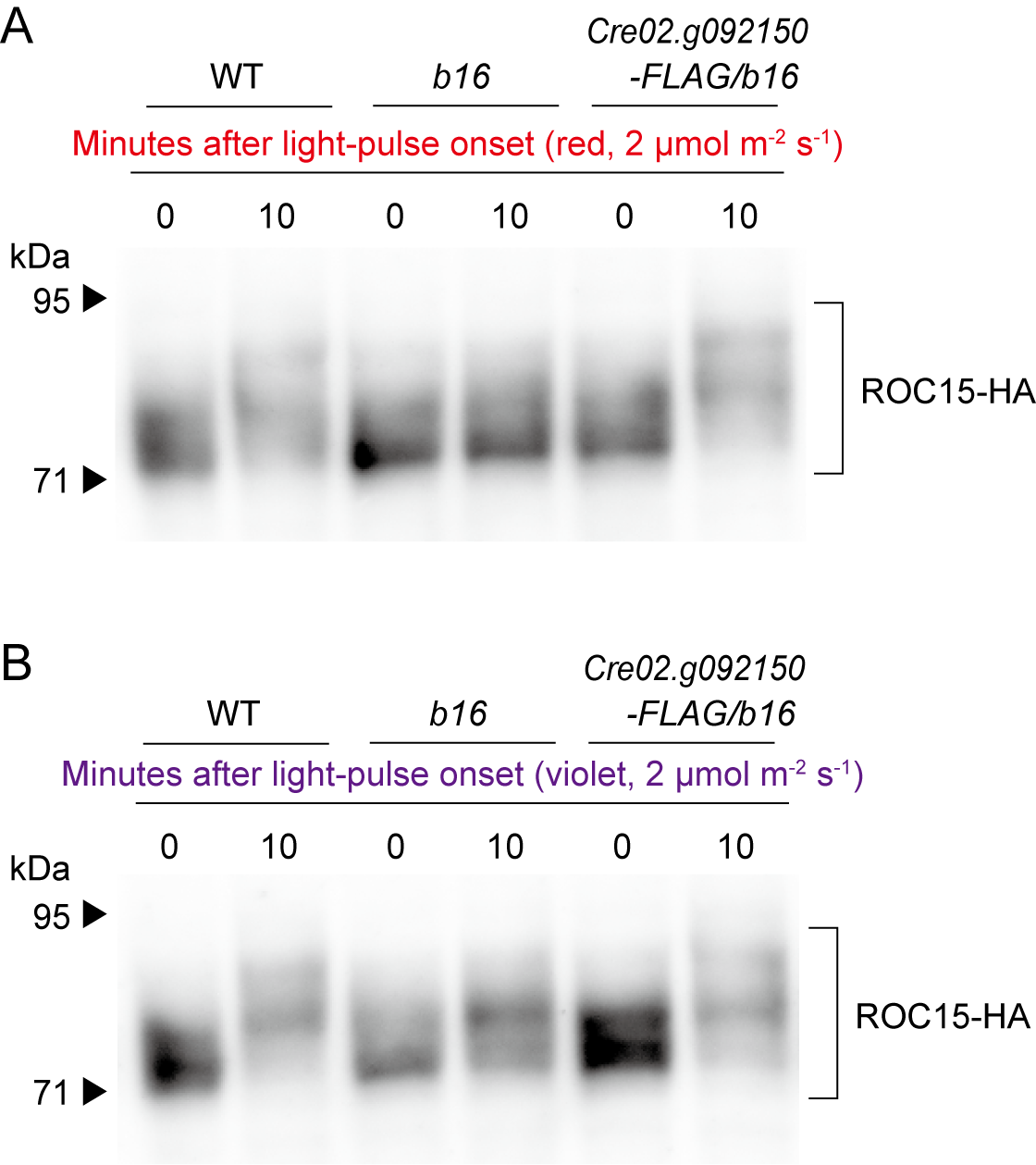

Supplement: S11 Fig — The Cre02.g092150-FLAG strain was crossed with the ROC15-HA strain [21], and progenies harboring the ROC15-HA and both the b16 mutation and Cre02.g092150-FLAG transgenes (Cre02.g092150-FLAG/b16) were selected by spot tests for antibiotic resistance and genotyping by genomic PCR. Cell cultures were prepared as described in Fig 4B, and exposed to a 0.5 min pulse of red light (660 nm, 2 μmol∙m-2∙s-1) or violet light (405 nm, 2 μmol∙m-2∙s-1) at midnight. Total protein extracts were subjected to Western blot analysis using anti-HA antibody. (TIF) [file pgen.1006645.s011.tif]

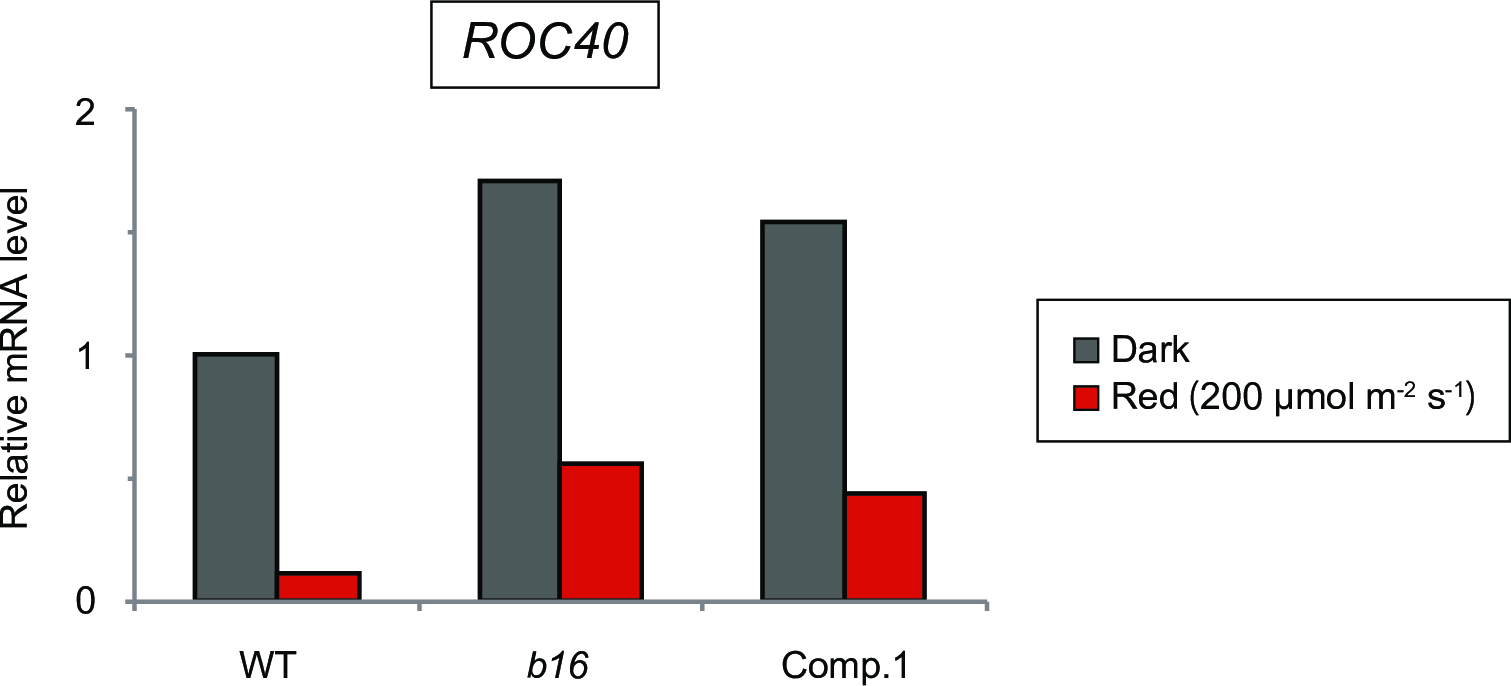

Supplement: S12 Fig — Samples of red light exposed cells were prepared as described in Fig 5. RT-qPCR results of ROC40 of the WT, b16, and the complemented strain (Comp1; Fig 6B, S6 Fig) are shown. The transcript abundances relative to RCK1 were further normalized by dividing by the dark control level of the WT for easy comparison. (TIF) [file pgen.1006645.s012.tif]

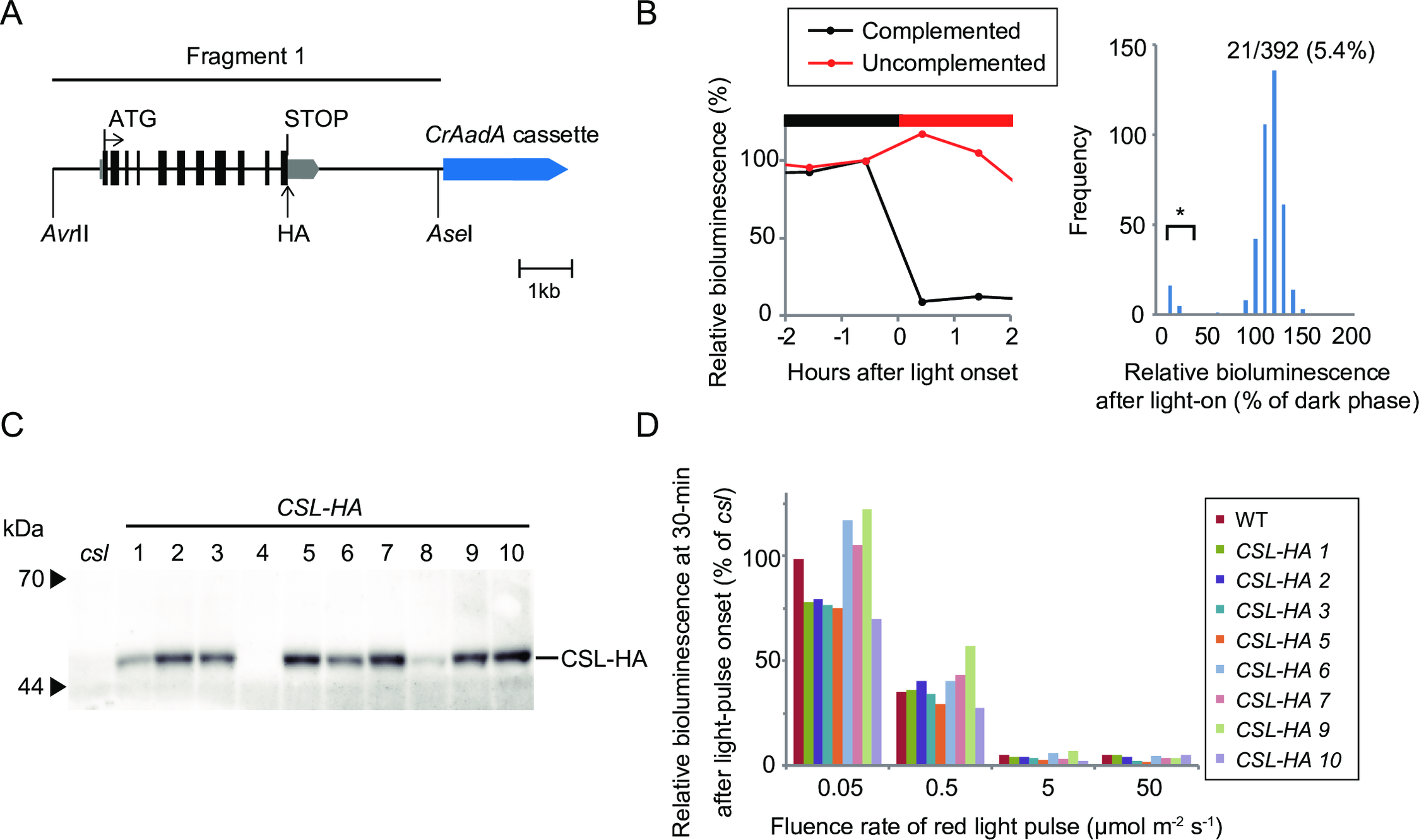

Supplement: S13 Fig — (A) Schematic representation of the CSL-HA gene. Black and gray boxes represent the CDS and UTRs, respectively. (B) Complementation of the csl phenotype by CSL-HA. csl mutant was transformed with CSL-HA gene. Data are collected and shown as Fig 6B, 6C and 6D. (C) Western blot analysis of CSL-HA. Cells of asynchronous TAP liquid cultures of the complemented strains were harvested and subjected to Western blot analysis with an anti-HA antibody. A protein sample of csl mutant was loaded as a negative control. (D) Light dose dependency of the light response of ROC15-LUC in CSL-HA strain. Asynchronous cultures of the CSL-HA strains were subjected to darkness for 3 h for accumulation of ROC15-LUC, and then 2 min red light pulses of indicated intensities were administered. Data indicated are relative bioluminescence levels of CSL-HA-expressed strains against that of the csl mutant 30 min after light pulse onset. (TIF) [file pgen.1006645.s013.tif]

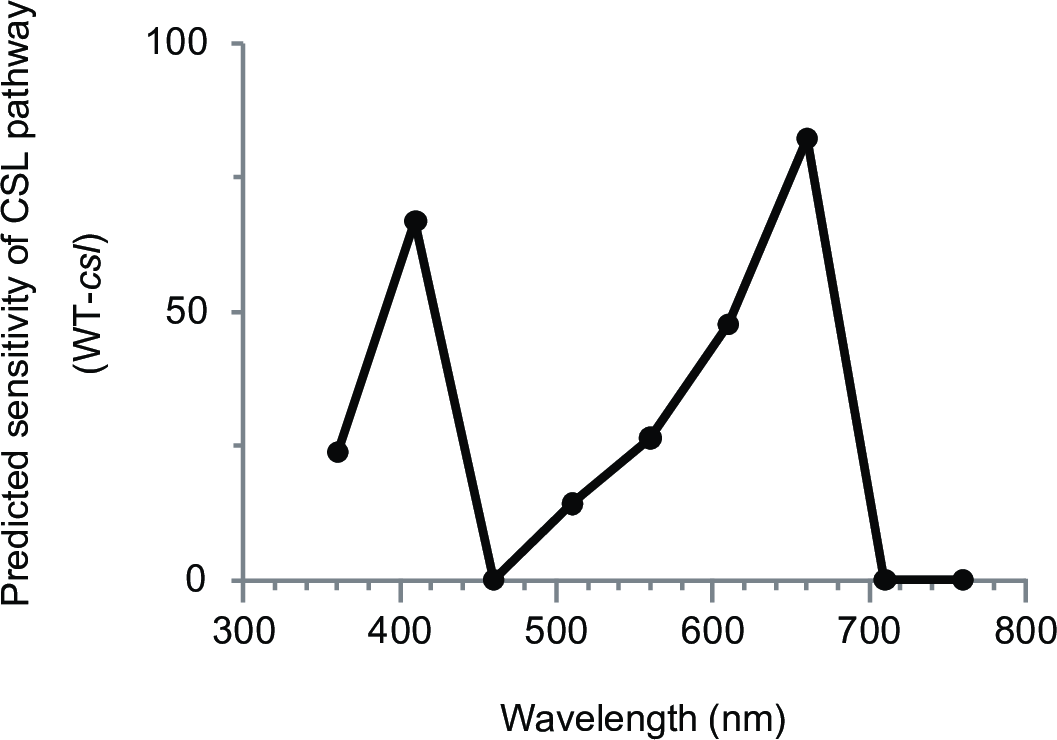

Supplement: S14 Fig — The sensitivity of ROC15 light response remaining in csl was subtracted from that of WT. Negative values were regarded as 0. (TIF) [file pgen.1006645.s014.tif]

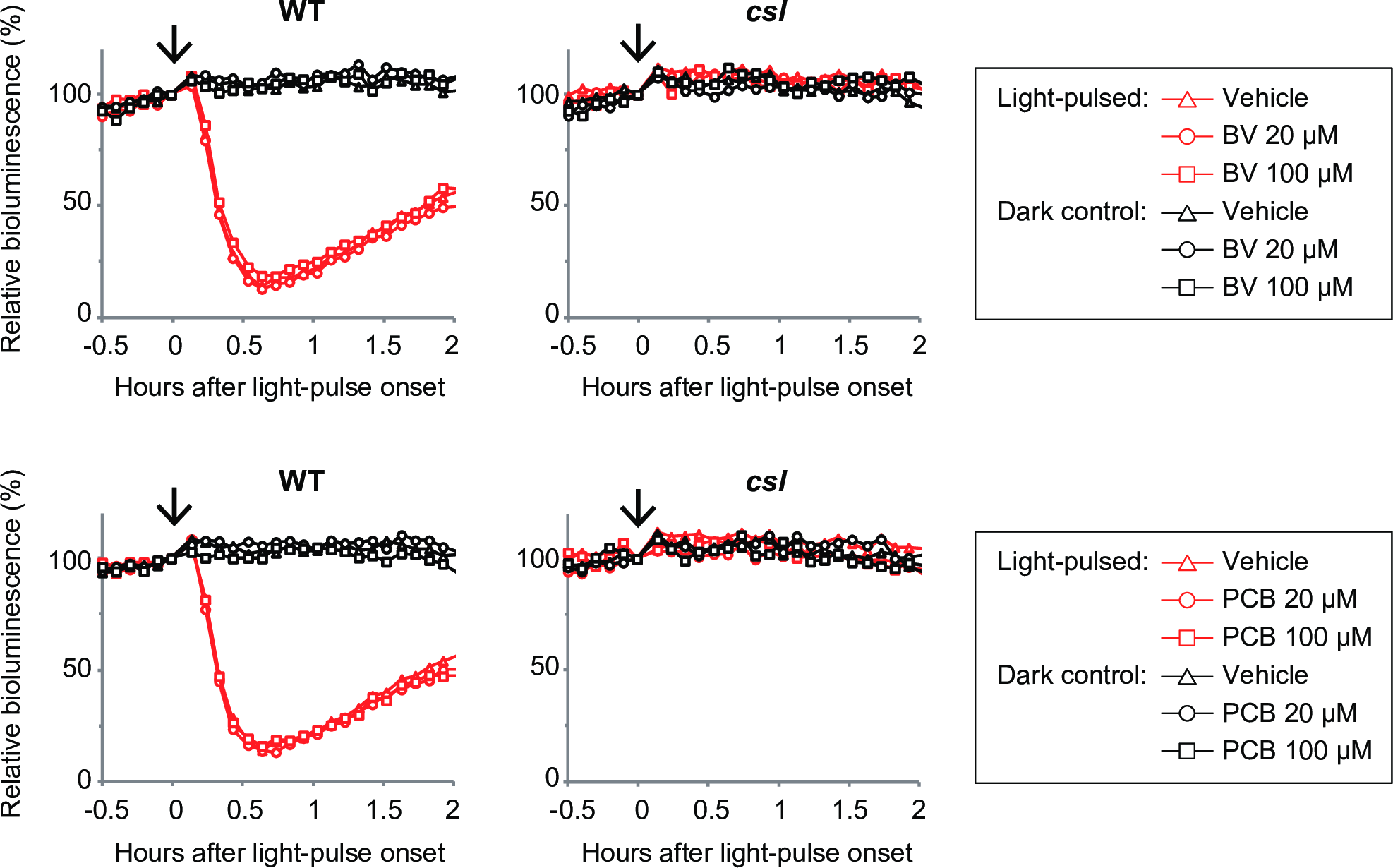

Supplement: S15 Fig — Asynchronous TAP cultures of WT and csl in black 24-well plates were subjected to darkness for 3 h for accumulation of ROC15-LUC, and then a red light pulse (2 μmol∙m-2∙s-1) were administered for 0.5 min (arrows). Billins (Frontier Scientific, UT, USA) were added to the cultures just before dark adaptation at the final concentrations indicated in the graphs. Each trace is the mean of bioluminescence from two independent cultures. The bioluminescence level just before light pulse was set to 100. (TIF) [file pgen.1006645.s015.tif]

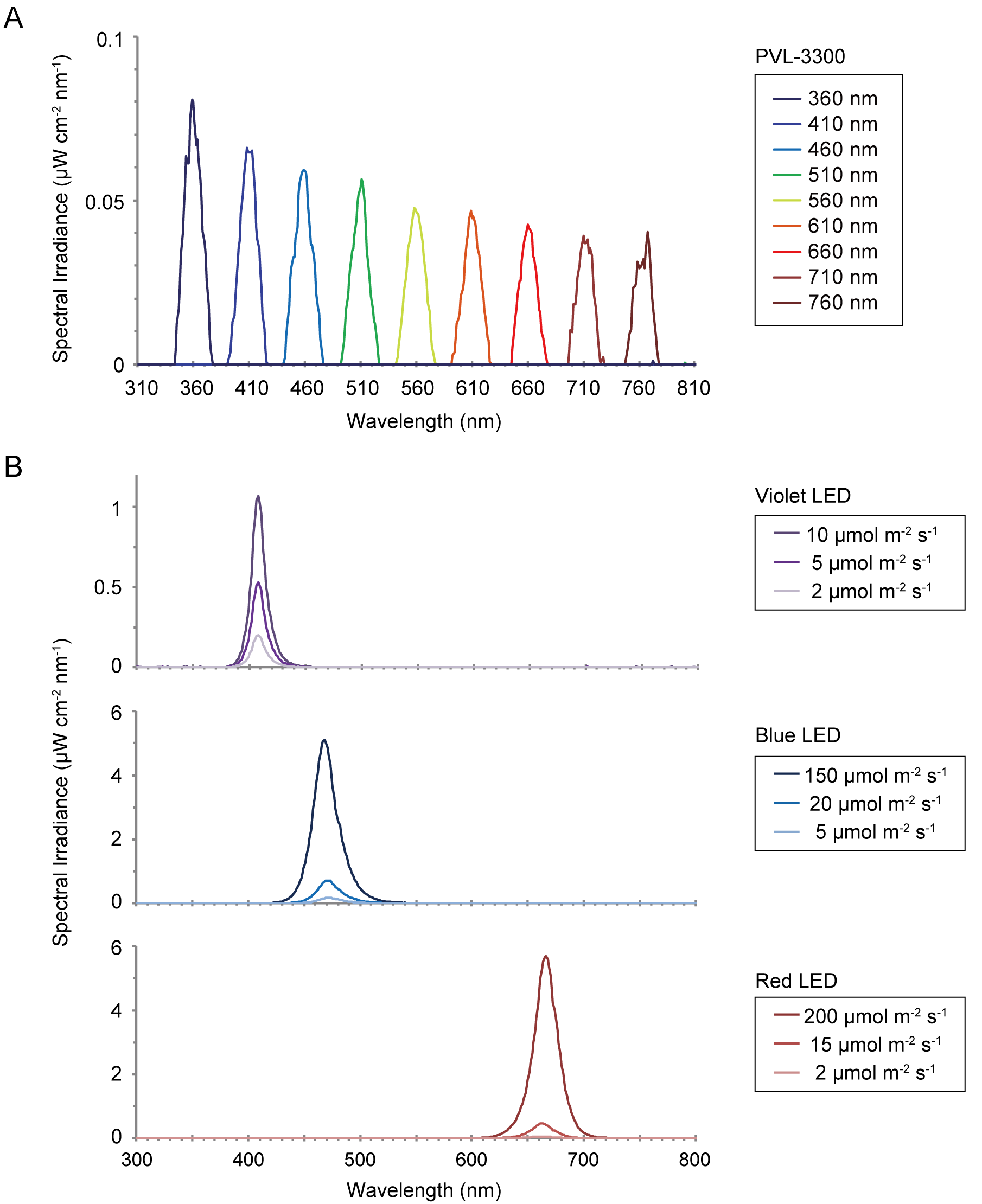

Supplement: S16 Fig — (A) Spectrograms of monochromatic light emitted by the tunable monochromatic light source PVL-3300 (Asahi Spectra, Tokyo, Japan). (B) Emission spectra of the violet, blue, and red LED (ISL-150×150-VV, ISL-150×150-BB, and ISL-150×150-RR, respectively; CCS, Kyoto, Japan). (TIF) [file pgen.1006645.s016.tif]
